# Signaling transcript profile of the asexual intraerythrocytic development cycle of *Plasmodium falciparum* induced by melatonin and cAMP

**DOI:** 10.18632/genesandcancer.118

**Published:** 2016-09

**Authors:** Wânia Rezende Lima, Giulliana Tessarin-Almeida, Andrei Rozanski, Kleber S. Parreira, Miriam S. Moraes, David C. Martins, Ronaldo F. Hashimoto, Pedro A.F. Galante, Célia R.S. Garcia

**Affiliations:** ^1^ Departamento de Fisiologia, Instituto de Biociências, Universidade de Sao Paulo, Sao Paulo, Brazil; ^2^ Centro de Oncologia Molecular, Hospital Sírio-Libanês, Sao Paulo, Brazil; ^3^ Departamento de Imunologia e Parasitologia, Instituto de Ciências Biomédicas, Universidade Federal de Uberlândia, Brazil; ^4^ Centro de Matemática, Computação e Cognição, Universidade Federal do ABC, São Paulo, Brazil; ^5^ Departamento de Ciência da Computação, Instituto de Matemática e Estatística, Universidade de São Paulo, São Paulo, Brazil; ^6^ Instituto de Ciências Exatas e Naturais (ICEN)- Medicina, Universidade Federal do Mato Grosso - Campus Rondonópolis, Brazil

**Keywords:** transcripts, malaria, *P. falciparum*, melatonin, cAMP, ubiquitin proteasome

## Abstract

According to the World Health Organization (WHO), *Plasmodium falciparum* is the deadliest parasite among all species. This parasite possesses the ability to sense molecules, including melatonin (MEL) and cAMP, and modulate its cell cycle accordingly. MEL synchronizes the development of this malaria parasite by activating several cascades, including the generation of the second messenger cAMP. Therefore, we performed RNA sequencing (RNA-Seq) analysis in *P. falciparum* erythrocytic stages (ring, trophozoite and schizont) treated with MEL and cAMP. To investigate the expression profile of *P. falciparum* genes regulated by MEL and cAMP, we performed RNA-Seq analysis in three *P. falciparum* strains (control, 3D7; protein kinase 7 knockout, PfPK7-; and PfPK7 complement, PfPK7C). In the 3D7 strain, 38 genes were differentially expressed upon MEL treatment; however, none of the genes in the trophozoite (T) stage PfPK7- knockout parasites were differentially expressed upon MEL treatment for 5 hours compared to untreated controls, suggesting that PfPK7 may be involved in the signaling leading to differential gene expression. Moreover, we found that MEL modified the mRNA expression of genes encoding membrane proteins, zinc ion-binding proteins and nucleic acid-binding proteins, which might influence numerous functions in the parasite. The RNA-Seq data following treatment with cAMP show that this molecule modulates different genes throughout the intraerythrocytic cycle, namely, 75, 101 and 141 genes, respectively, in the ring (R), T and schizont (S) stages. Our results highlight *P. falciparum*'s perception of the external milieu through the signaling molecules MEL and cAMP, which are able to drive to changes in gene expression in the parasite.

## INTRODUCTION

Malaria is a major cause of death worldwide, and *Plasmodium falciparum* is the species responsible for the majority of malaria cases. In mammals, the parasite *P. falciparum* has adapted to living in red blood cells during its asexual life cycle. Our previous work showed that the host circadian rhythm, which is regulated by melatonin (MEL), a molecule produced by the pineal gland in response to darkness [1], plays an important role in controlling proliferation and synchronizing the development of the malaria parasite [2, 3]. This regulation is achieved by activating signaling cascades, including However, how these parasites respond to regulation by the host molecules MEL and cAMP, which modulate the proliferation cycle *in vitro*, is still unknown. It is becoming increasingly clear that chromatin remodeling and epigenetic memory play an important role in the the PLC-IP pathway; cross-talking; and generating the expression and antigenic variation of virulence genes in second messengers cAMP and IP, which elicit an increase in cytosolic calcium. As to the molecular machinery involved, these components could include proteases, kinases and genes that belong to the UPS [4, 5]. More strikingly, the protein kinase 7 (PK7) knockout strain PfPK7 is unable to respond to MEL and to synchronize the intraerythrocytic forms of the parasites [6].

In recent decades, much effort has been focused on understanding how gene regulation is achieved in *Plasmodium*. Unfortunately, although the *P. falciparum* genome sequencing is complete [7], half of the genes remain without annotation; therefore, understanding parasite biology poses a large challenge. Microarray technology was used to obtain information regarding the transcriptome of the parasite during its sexual development [8] and during the intraerythrocytic developmental cycle [9] to profile the malaria parasite in its different life stages [10]. Moreover, high-throughput sequencing of cDNA (RNA-Seq) presented a new opportunity to deeply probe the *Plasmodium* transcriptome. RNA-Seq analyses allowed the detection of novel gene transcripts, the correction of a large number of genes, and the refinement of the original gene model, revealing tight regulation of gene expression throughout the intraerythrocytic development cycle of *P. falciparum* [11–16].

The mechanisms for controlling the cell cycle and development in malaria parasites are far from being fully understood. Dissecting the cellular signaling networks is an important goal for not only understanding the biology of these parasites but also providing new ways to combat malaria infection [17].

In the last decade, studies from several labs considerably advanced our understanding of how the organisms in the genus *Plasmodium* sense the signaling milieu and trigger their cellular and molecular machinery involved in transducing these signals [4, 6, 18–21].

Recently, cAMP has been identified as a key regulator that triggers the timely secretion of microneme proteins, enabling receptor engagement and invasion [22]. This study also observed that cAMP increases merozoite cytosolic Ca2+ levels via induction of the Epac pathway; together, they are involved in the invasion process [22]. Moreover, our group reported that the cAMP analog adenosine 3′,5′-cyclic monophosphate N6-benzoyl/PKA activator (6-Bz-cAMP) is able to increase the schizont (S) stage, leading to progression of the asexual stage of the life cycle and modulation of PFNF-YB transcription factor expression during the intraerythrocytic cycle in *P. falciparum* [23].

However, how these parasites respond to regulation by the host molecules MEL and cAMP, which modulate the proliferation cycle *in vitro*, is still unknown. It is becoming increasingly clear that chromatin remodeling and epigenetic memory play an important role in the expression and antigenic variation of virulence genes in infected red blood cells (iRBCs) [24–26].

We generated RNA-Seq data to study *P. falciparum*- infected RBCs at the trophozoite (T) stage of the wild-type (WT) strain (3D7), the PK7 knockout strain (PfPK7-) and its complement strain (PfPK7C) to determine the target genes involved in parasite synchronization by MEL. This approach also helped us to identify possible targets and the role of PK7 in gene transcription. The results revealed that incubation of *P. falciparum-*infected cells with MEL for 5 hours led to considerable changes in gene expression in the 3D7 strain compared to that of the control. When similar experiments were performed in PfPK7 knockout strains, we did not observe any differential gene expression in MEL treated and untreated assays. These findings are strong evidence of the relevance of this gene (PfPK7) in MEL-induced regulation of the cell cycle. To better understand the signaling cascade activated by cAMP, we treated ring (R), T and S stage parasites with the cAMP analog 6-Bz-cAMP. Our results showed that cAMP and MEL treatment affected the mRNA transcript levels of UPS genes at the T stage. In addition to the modulation of kinases and transcription factors, cAMP treatment altered the expression of ribosomal proteins at the R stage and of isomerase, rotamase and binding proteins involved in metabolic processes, such as coenzymes, at the S stage.

## RESULTS

### Sequencing and data processing

We previously reported that a subset of genes related to the ubiquitin proteasome system (UPS) was modulated in *P. falciparum* after 5 hours of treatment with MEL [6]. Therefore, we performed RNA-Seq analysis with synchronized parasites in three *P. falciparum* strains (control 3D7, protein kinase 7 knockout, PfPK7-, and PfPK7 complementPfPK7C).

We collected total RNA from synchronized T stage 3D7 parasites that were treated with MEL (final concentration, 100 nM) and incubated for 5 hours at 37°C. Total RNA samples were processed as described below (see Methods and Supplementary Figure 1). Every treatment and RNA-Seq were performed on two biological replicates. In summary, ~81.6% (3D7), ~79% (PfPK7-) and ~81.5% (PfPK7C) of reads were mapped uniquely against the *Plasmodium* genome to verify the sequencing quality (Supplementary Tables 1 and 2).

### Melatonin treatment leads to a change in *P. falciparum* gene expression

**Figure 1:**
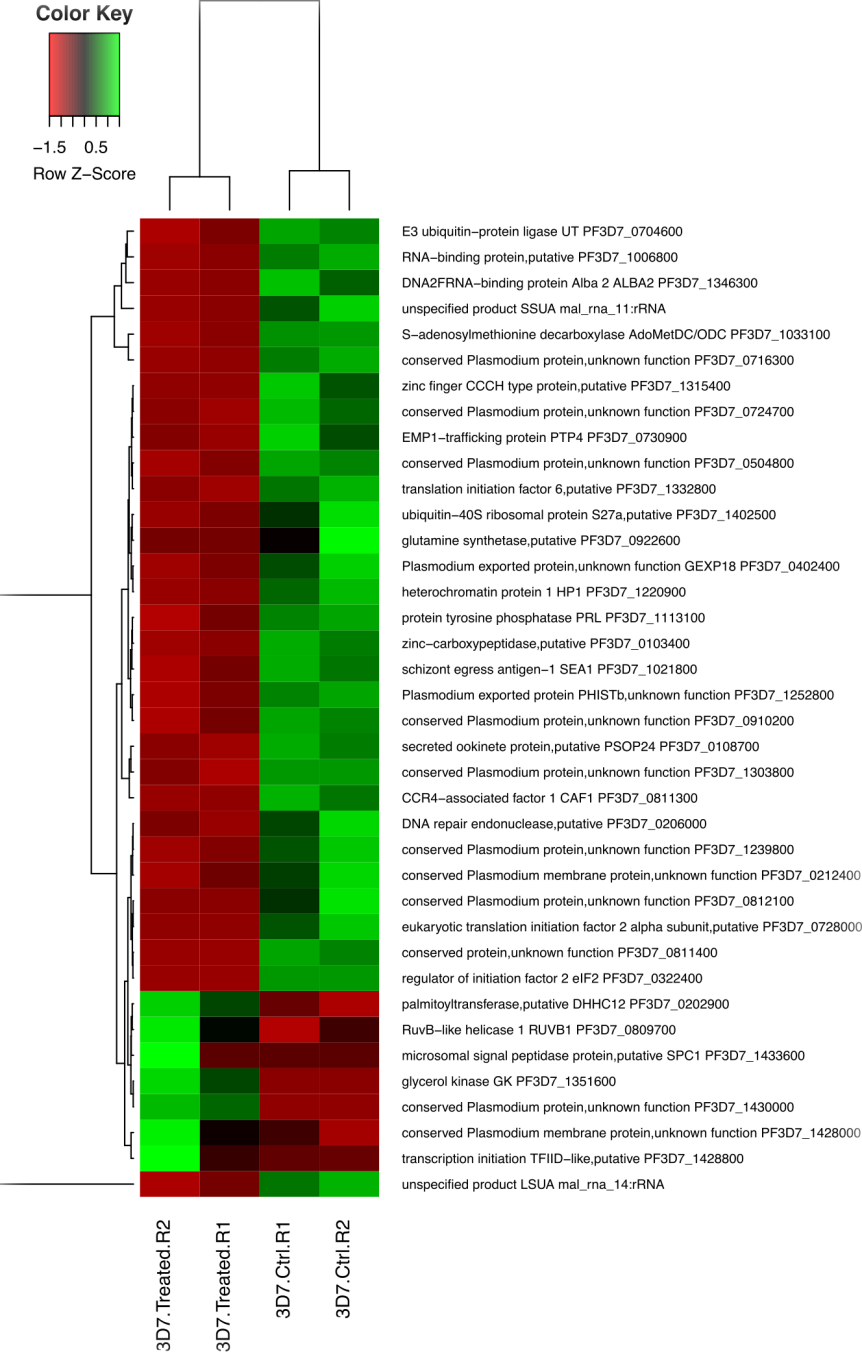
Differentially expressed genes detected in the trophozoite stage of the *P. falciparum* 3D7 strain treated with melatonin for 5 hours Heat map of 38 differentially expressed genes affected by melatonin treatment. Gene expression in the trophozoite stage *P. falciparum* 3D7 parasites treated with 100 nM melatonin for 5 hours was compared with that of the same stage *P. falciparum* 3D7 parasites treated with ethanol as a control. R1 and R2 are duplicates.

Next, we compared the gene expression profiles of the *P. falciparum* parasite lines (3D7 and PfPK7- clones) untreated and treated with MEL at 37°C. The results revealed that 100 nM MEL caused a change in gene expression only for the 3D7 strain after 5 hours of incubation. Among the 38 differentially expressed genes in the MEL-treated 3D7 strain, 7 genes were down-regulated and 31 genes were up-regulated (p-value < 0.05; FDR < 0.05) compared with the treated and untreated 3D7 strains (Supplementary Table 3 and Figure 1).

The differentially expressed genes up-regulated by the MEL treatment included E3 ubiquitin ligase (PF3D7_0704600); regulator of initiation factor 2 (eIF2; PF3D7_0322400), which plays an important role in post- transcriptional control of gene expression in malaria parasites; and the gene encoding S-adenosylmethionine decarboxylase/ornithine decarboxylase (AdoMetDC/ ODC; PF3D7_1033100).

The MEL treatment also up-regulated a series of genes related to RNA metabolism, including a zinc finger protein (PF3D7_0202900), zinc finger (CCCH type) protein (PF3D7_1315400), translation initiation factor 6 (PF3D7_1332800), eukaryotic translation initiation factor 2 alpha subunit (PF3D7_0728000), transcription initiation TFIID-like (PF3D7_1428800), DNA/RNA-binding protein Alba 2 (ALBA2; PF3D7_1346300), RNA-binding protein (PF3D7_1006800), DNA repair endonuclease (PF3D7_0206000), ubiquitin-40S ribosomal protein S27a (PF3D7_1402500), and heterochromatin protein 1 (PF3D7_1220900).

Additionally, the gene encoding CCR4-Associated Factor 1 (CAF1) was up-regulated after the MEL treatment. CAF1 coordinates the expression of *P. falciparum* egression and invasion proteins, which are extremely important for asexual blood-stage development [27].

Among the seven differentially expressed genes down-regulated by the MEL treatment, we found microsomal signal peptidase (PF3D7_1433600), which has an important role in the transport and maturation of parasite proteins; glycerol kinase (GK; PF3D7_1351600), which is required for optimal intraerythrocytic asexual parasite development; SPC1 (PF3D7_1433600) and RUVBL31 (PF3D7_0809700).

**Figure 2:**
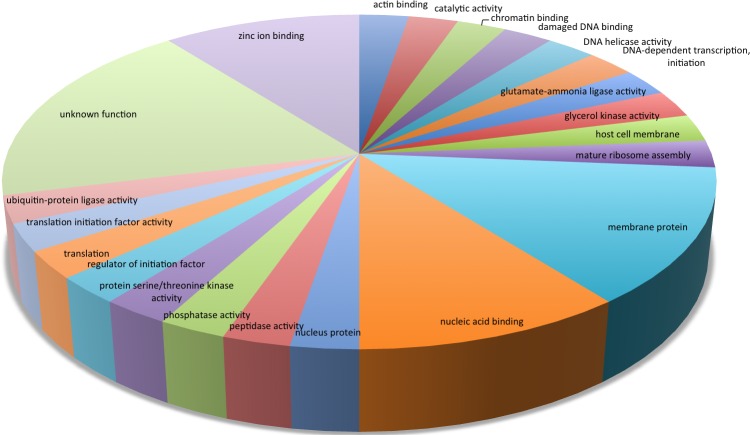
Pie chart showing the distribution of GO categories for the 38 differentially expressed genes in the 3D7 strain after 5 hours of incubation with 100 nM melatonin

Next, we identified the gene function of these up- and down-regulated genes. Figure 2 is a pie chart showing the distribution of Gene Ontology (GO) [28] terms for the 38 differentially expressed genes in the 3D7 strain. Using GO annotation, we found that 4 of 11 genes that transcribe conserved *Plasmodium* proteins with unknown function are annotated as genes coding for membrane proteins, one gene is annotated as a gene coding for a nuclear protein, one gene is annotated as a gene coding for protein serine/threonine kinase activity, one gene is annotated as a gene coding for actin binding, and one gene is annotated as a gene coding for zinc ion binding. In total, 29% of the differentially expressed genes (11 genes) had no functional annotation.

### cAMP stimulates cell division and represses cell host invasion gene expression in the trophozoite stage of *P. falciparum*

**Figure 3:**
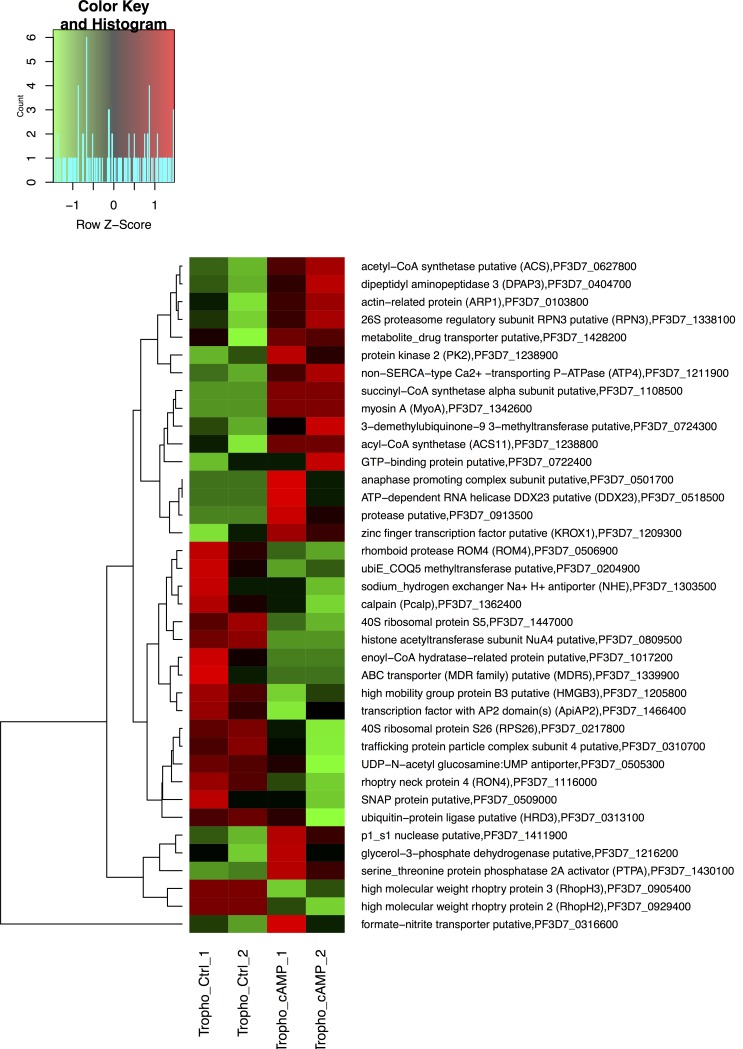
Differentially expressed genes detected in the trophozoite stage of the *P. falciparum* 3D7 strain treated with cAMP for 6 hours Heat map of 38 differentially expressed genes affected by exposing trophozoite stage *P. falciparum* 3D7 parasites to 20 μM 6-Bnz-cAMP analog compared to the non-treated 3D7 trophozoite parasites. Only those genes that had a fold change in expression > 2 are displayed.

The second messenger cAMP participates in several biological activities in eukaryotic cells and interplays with the MEL pathway [5, 29]. Because 6-Bz-cAMP is able to modulate the *P. falciparum* life cycle by increasing the number of parasites at the S stage, thus leading to progression of the asexual stage of the life cycle, we investigated the role that cAMP plays during the asexual intraerythrocytic cycle of *P. falciparum.* Highly synchronous trophozoites at the 34-hours timepoint were treated with 6-Bz-cAMP compound (Supplementary Figure 2). Every treatment and RNA-Seq were performed on two biological replicates. At the T stage, we found that 49 genes were up-regulated, and 52 genes were down- regulated (Figure 3 and Supplementary Table 4). We used the Database for Annotation, Visualization and Integrated Discovery (DAVID) tool to search for genes involved in the cAMP pathway in *P. falciparum* [30, 31]. Eighty-one DAVID gene IDs were identified, but only six chart records were found. The analysis revealed that three of six pathways noted from the functional annotation were significantly modified by cAMP (see Table 1).

**Table 1:** Pathways enriched in *P. falciparum* treated with cAMP as identified by functional annotation cluster analysis using a chart from the DAVID Bioinformatics database

	Ring stage		
Annotation cluster	Enrichment score: 1.16	Count	p-value
SP_PIR_KEYWORDS	ribosomal protein	7	0.0048
GOTERM_MF_FAT	structural constituent of ribosome	7	0.011
GOTERM_BP_FAT	translation	9	0.011
KEGG_PATHWAY	ribosome	6	0.012
GOTERM_MF_FAT	structural molecule activity	7	0.026
GOTERM_CC_FAT	ribosome	7	0.047
	**Trophozoite stage**		
Annotation cluster	Enrichment score: 0.38		
GOTERM_CC_FAT	Golgi apparatus	3	0.042
KEGG_PATHWAY	ubiquinone and other terpenoid-quinone biosynthesis	2	0.042
GOTERM_BP_FAT	quinone cofactor metabolic process	2	0.046
	**Schizont stage**		
Annotation cluster 1	Enrichment score: 1.32		
SP_PIR_KEYWORDS	isomerase	5	0.0094
SP_PIR_KEYWORDS	rotamase	3	0.031
INTERPRO	peptidyl-prolyl cis-trans isomerase, cyclophilin-type	3	0.031
GOTERM_MF_FAT	peptidyl-prolyl cis-trans isomerase activity	3	0.047
GOTERM_MF_FAT	cis-trans isomerase activity	3	0.047
Annotation cluster 2	Enrichment score: 1.17		
GOTERM_BP_FAT	coenzyme metabolic process	5	0.025
GOTERM_MF_FAT	acid-amino acid ligase activity	4	0.026

### cAMP signaling targets different genes during the intraerythrocytic cell cycle of *P. falciparum*

We also investigated the cAMP signaling targets at the R and S stages. To achieve this goal, we performed RNA-Seq using samples from highly synchronized iRBCs. The parasite strain 3D7 was synchronized in R and S forms. After the iRBCs were synchronized, they were selected at 6 hours and 44 hours for treatment with 20 μM of a cAMP analog for 6 hours at 37°C. The genome- wide transcriptome analysis of the R and S stages revealed 75 and 141 genes, respectively, that were differentially expressed based on fold changes greater than 2.0 or lower than −2.0, CPM ≥ 1, and a p-value < 0.05.

**Figure 4:**
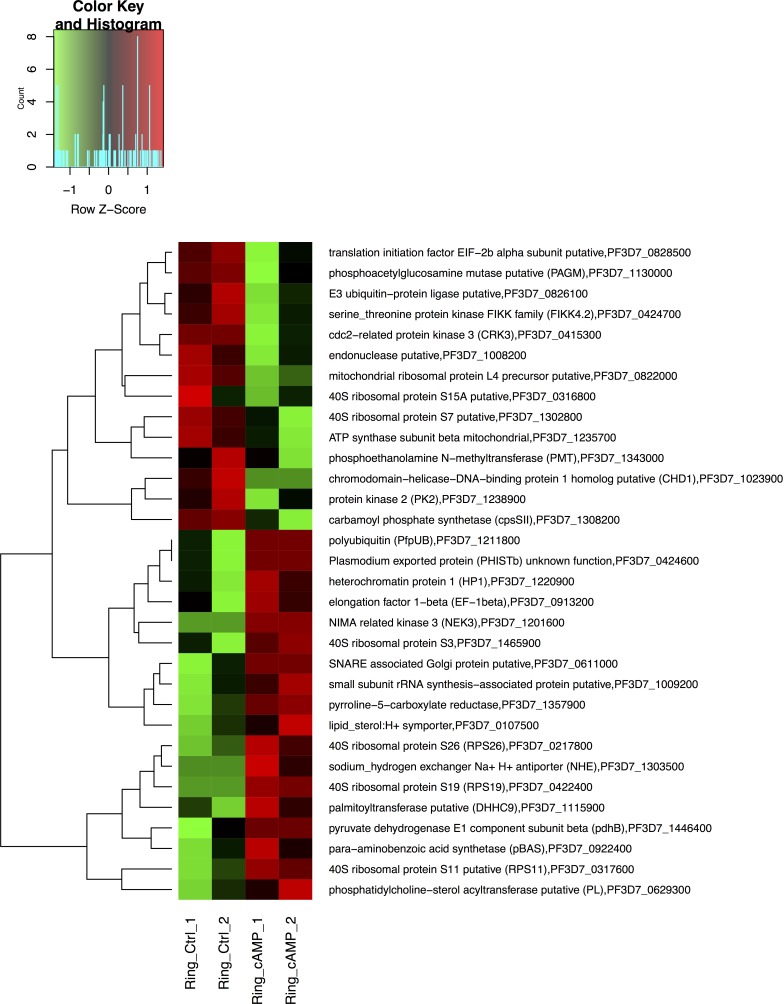
Differentially expressed genes detected in the ring stage of the *P. falciparum* 3D7 strain treated with cAMP for 6 hours Heat map of 32 differentially expressed genes affected by treating ring stage *P. falciparum* 3D7 parasites with 20 μM 6-Bnz-cAMP compound compared to non-treated 3D7 ring parasites. Only those genes that had a fold change in expression > 2 are displayed.

To understand the role of cAMP during the R stage, analyses of the whole transcripts modified following cAMP analog treatment were performed. We identified 36 up-regulated genes and 39 down-regulated genes (Figure 4). Using the DAVID bioinformatics database, 66 DAVID gene IDs were functionally annotated. Three clusters of biological activities were formed, and one group of ribosomal genes presented a significant p-value (Table 1). The genes in this cluster were found to be related to ribosomal structure and function as identified by GO term annotation and KEGG pathway analysis. These genes were classified in (a) ribosomal protein, (b) structural constituent of ribosome, (c) translation, (d) ribosome and (e) structural molecule activity. KEGG pathway analysis identified a variety of up-regulated genes involved in structural constituents of ribosomes, such as small subunit ribosomal proteins RP-S11e, RP-S19e, RP-S26e, and RP-S3e (PF3D7_0317600/ K02949, PF3D7_0422400/K02966, PF3D7_0217800/K02976 and PF3D7_1465900/K02985); biosynthesis of secondary metabolites and antibiotics, such as pyruvate dehydrogenase E1 component beta subunit and pyrroline-5-carboxylate reductase (PF3D7_1446400/K00162 and PF3D7_1357900/K00286). By contrast, several ATP- binding proteins were found to be down-regulated upon cAMP treatment: chromodomain-helicase-DNA-binding protein 1 homolog, putative (CHD1; PF3D7_1023900); protein kinase 2 (PK2; PF3D7_1238900); carbamoyl phosphate synthetase (cpsSII, PF3D7_1308200); a conserved *Plasmodium* protein of unknown function (PF3D7_0315400); serine/threonine protein kinase, FIKK family (FIKK4.2; PF3D7_0424700), cdc2-related protein kinase 3 (CRK3; PF3D7_0415300); and ATP synthase subunit beta, mitochondrial (PF3D7_1235700). For additional details, see Supplementary Table 5.

**Figure 5:**
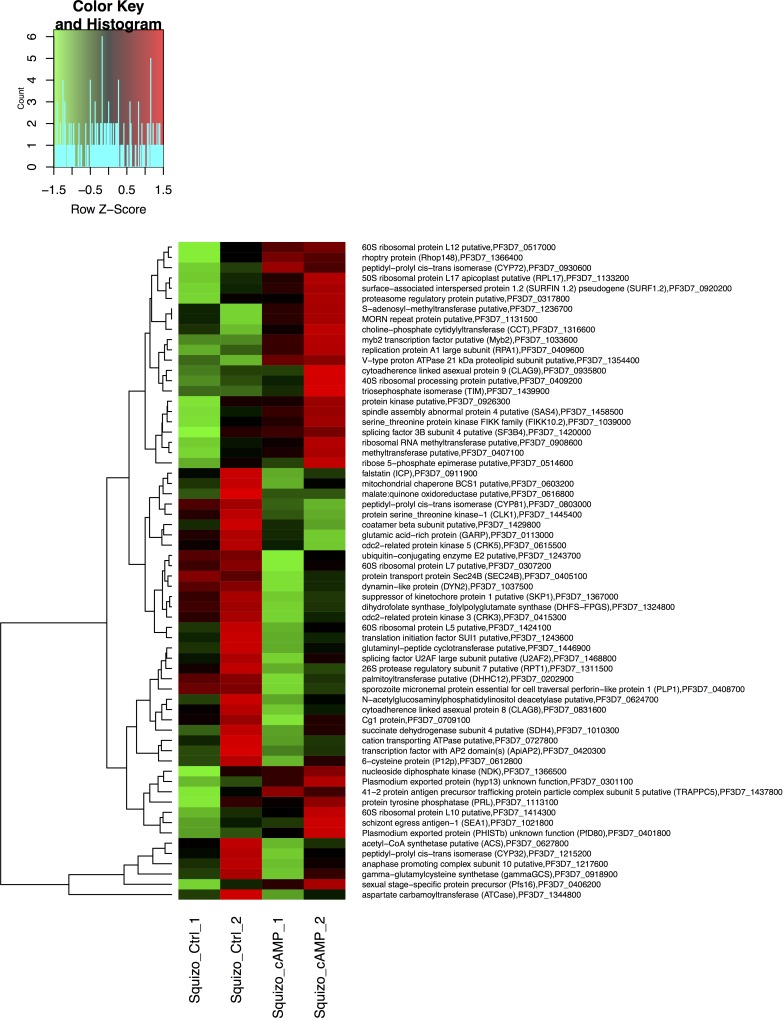
Differentially expressed genes detected in the schizont stage of *P. falciparum* 3D7 strain treated with cAMP for 6 hours Heat map of 64 differentially expressed genes affected by exposing schizont stage *P. falciparum* 3D7 parasites to 20 μM 6-Bnz- cAMP compound compared to non-treated 3D7 schizont parasites. Only those genes that had a fold change in expression > 2 are displayed.

The DAVID bioinformatics database was used to analyze the differentially expressed genes regulated by cAMP treatment during the S stage. In total, 132 DAVID gene IDs matched 141 genes identified as differentially expressed by RNA-Seq. Functional annotation generated ten clusters, and only two clusters presented a significant p-value (Table 1). Cluster 1 represented isomerase activity, and cluster 2 represented coenzyme metabolic process and acid-amino acid ligase activity genes. Among the genes coding for isomerases and rotamases, cAMP modulated genes coding for the following peptidyl-prolyl cis-trans isomerases: CYP81 (PF08_0128), CYP72 (PFI1490c), and CYP32 (PFL0375w) and triosephosphate isomerase (TIM; PF14_0378). In cluster 2, coenzyme metabolic process was modulated by mRNA expression of dihydrofolate synthase/folylpolyglutamate synthase (DHFS-FPGS; PF13_0140), glutathione synthetase (GS; PFE0605c), ribose 5-phosphate epimerase (PFE0730c), and gamma- glutamylcysteine synthetase (gammaGCS; PFI0925w). Furthermore, the following genes involved in acid-amino acid ligase activity were down-regulated by cAMP (Figure 5): DHFS-FPGS (PF13_0140), GS (PFE0605c), gammaGCS (PFI0925w) and ubiquitin-conjugating enzyme E2 (PFL2100w).

We also observed a series of up- and down- regulated kinases (Figure 5 and Supplementary Table 6). Many *Plasmodium* kinases have recently been shown by reverse genetics to be crucial for various parts of the complex parasitic life cycle and are therefore potential targets [32]. Among the up-regulated kinases, we would like to highlight serine/threonine protein kinase, FIKK family (FIKK10.2; PF3D7_1039000); protein kinase (PF3D7_0926300); and nucleoside diphosphate kinase (NDK; PF3D7_1366500).

Recently, data mining of the *P. falciparum* genome revealed a new putative kinase family, which was called the FIKK family because of the conservation of a phenylalanine, isoleucine, lysine, and lysine amino acid motif [33–35]. The FIKK gene is located at chromosomal subtelomeric regions near several other differentially expressed genes that are important for remodeling the intraerythrocytic membrane [36]; consequently, FIKK could be involved in specific host-parasite interactions, such as the regulation of expression of the var gene family [36].

Nucleoside diphosphate kinase plays a crucial role in maintaining the intracellular energy resources and balancing nucleotide pools. [37]. Nucleoside diphosphate kinase plays a unique metabolic role, as this enzyme performs the last reaction of ribonucleotide and deoxyribonucleotide biosynthesis. This enzyme was found to bind a broad range of ribo- and deoxyribonucleotides; thus, it can produce all precursors of DNA and RNA synthesis. NDK produces NTP for nucleic acid synthesis, CTP for lipid synthesis, UTP for polysaccharide synthesis and GTP for protein synthesis, signal transduction or microtubule polymerization [38].

Among the down-regulated kinases, we highlight protein serine/threonine kinase-1 (CLK1; PF3D7_1445400), cdc2-related protein kinase 3 (CRK3; PF3D7_0415300) and cdc2-related protein kinase 5 (CRK5; PF3D7_0615500). CLK1 is part of the CMGC group of kinases, which interact with factors involved in mRNA splicing. As previously described, PfLAMMER (PF14_0431) [39] is related to yeast kns1 [40] and the human LAMMER kinases CLK1-4, which also phosphorylate SR proteins [41]. CRK3 encodes an uncommonly large CDK-related protein (1,339 amino acids) whose kinase domain displays maximal homology to those CDKs, and this protein is involved in the control of transcription in other eukaryote organisms [35]. It has been hypothesized that PfCRK-3 is part of a complex containing other protein kinases because this enzyme is associated with a kinase activity present in the parasite extracts. Moreover, it was previously demonstrated that Pfcrk-3 interacts with a histone deacetylase (HDAC) in parasite extracts, suggesting that the pfcrk-3 gene plays a crucial role in parasite proliferation during the asexual erythrocytic cycle [35].

## DISCUSSION

Decoding how transcription is modulated in *P. falciparum* is fundamental to understanding parasite cell cycle regulation. MEL is a ubiquitous molecule that plays a central role in plants, mammals and parasites [42]. Here, we used the WT 3D7 and PfPK7- strains of *P. falciparum* to investigate transcriptional changes at various developmental stages upon MEL treatment. We attempted to complement the function of PfPK7 by episomal expression of the gene; however, this parasite strain did not show a complementation phenotype (data available at the online data repository) possibly due to incorrect timing of expression or low expression levels from the episomal plasmid. The identified E3 ligase (PF3D7_0704600) is likely part of a ubiquitin-mediated pathway that is triggered by an elegant activation and transfer cascade [43]. Once MEL and the UPS interact, gene expression of UPS members in *P. falciparum* is temporarily controlled [44](41); it is extremely important to identify these genes among the differentially expressed genes following MEL treatment. Interestingly, MEL and UPS also interact in different biological systems [45].

Another up-regulated gene is the gene encoding S-adenosylmethionine decarboxylase/ornithine decarboxylase (AdoMetDC/ODC; PF3D7_1033100). Polyamine synthesis is mainly under the control of the rate-limiting decarboxylases ODC (ornithine decarboxylase) and AdoMetDC (S-adenosylmethionine decarboxylase) [46](43). The intermolecular interactions between AdoMetDC and ODC seem to be vital for optimal ODC activity [47–49](44-46) and seem to be a viable strategy to aid in the design of selective inhibitors of polyamine metabolism of *P. falciparum* [50]*(47)*. The protein ALBA (Acetylation lowers binding affinity; PF3D7_1346300) is part of the DNA/RNA-binding protein family [51, 52](48,49). MEL treatment also causes the up-regulation of a gene encoding an RNA-binding protein. Recently, the protein encoded by this gene was identified as a homolog of Gbp2 [53]. Gbp2 seems to be expressed in all the developmental stages of the parasite (based on PlasmoDB expression data). The homolog of Gbp2, PfGbp2, is a small protein containing an arginine-rich region and two RNA recognition motifs (RRMs), one in the N-terminal and the other in the C-terminal region of the protein.

The proteins known as eukaryotic initiation factors (eIFs) play an important role in post-transcriptional control of gene expression in malaria parasites [54](52); their expression results in the selective translation of mRNAs encoding stress-response proteins. We observed up-regulation of the gene coding eIF2 (PF3D7_0322400) in the T stage parasites treated with MEL.

The *P. falciparum* heterochromatin protein 1 (PF3D7_1220900) was identified as a major structural component of virulence gene islands throughout the parasite genome; it binds specifically to a reversible histone modification, which marks these virulence loci for transcriptional silencing [55](53). Additionally, HP1 was identified as a protein that controls the antigenic variation of a protozoan parasite [56](54). MEL treatment up-regulated two genes coding for *Plasmodium* exported proteins GEXP18 (PF3D7_0402400) and PHISTb (PF3D7_1252800), which could indicate a unifying epigenetic strategy in the regulation of host-parasite interactions and immune evasion in *P. falciparum*.

The differentially expressed genes that were down- regulated by MEL treatment also included microsomal signal peptidase protein (PF3D7_1433600) and glycerol kinase (GK) (PF3D7_1351600). Signal peptidases are membrane-bound endopeptidases that have an important role in the transport and maturation of parasite proteins [57](55).

PfSEA1 (PF3D7_1021800), essential for blood- stage replication [58],(58) and protein tyrosine phosphate PfPRL (PF3D7_1113100), which plays a role in the merozoite stage, were positively modulated by MEL.

We investigated the effect of cAMP on the R, T, and S stages of the parasite life cycle, which lasts 48 hours. For each stage, we selected one point: R (6 hours), T (34 hours) and S (44 hours). Our results showed that cAMP could modulate several genes implicated in a variety of biological activities. Specifically, at the T stage following cAMP treatment for 6 hours, genes involved in cell division, proliferation, egress and UPS were up-regulated. Moreover, metabolic pathways such as fatty acid biosynthesis and degradation, spliceosome, peroxisome, citrate cycle and ubiquinone were positively modulated. The KROX1 protein is associated with proliferation [61], and the 26S proteasome regulatory subunit RPN3 and anaphase promoting complex subunit are crucial for UPS operation. Indeed, we believe that the cross-talk between MEL signaling and cAMP partially occurs through UPS proteins because they are also down-regulated by cAMP treatment. Examples of these UPS proteins include ubiquitin protein ligase, putative (HRD3) and ubiE/ COQ5 methyltransferase. MEL treatment augmented E3 ubiquitin ligase and ubiquitin-40S ribosomal protein S27a mRNA levels, showing that they did not regulate the same genes. However, cAMP and MEL share the same signaling pathway that controls protein activities and, consequently, the cell cycle and cell development [4, 5]; therefore, we presented these results in Table 2. MEL and cAMP modulate genes that are coupled to aerobic respiration and ATP production, such as S-adenosylmethionine decarboxylase/ornithine decarboxylase (AdoMetDC/ ODC), glutamine synthetase, succinyl-CoA synthetase alpha subunit and acyl-CoA synthetase (ACS11). These results may explain why the parasite replicates faster when treated with MEL and cAMP; the regulation of these groups of genes favors metabolic pathways, thus accelerating parasite maturation.

**Table 2:** Enriched pathways of *P. falciparum* treated with cAMP and melatonin

Ring (cAMP)				
Gene ID	Gene	Gene Description	Pathway	Fold Change*
**PF3D7_1211800**	2277.t00118, MAL12P1.117, PFL0585w	polyubiquitin (PfpUB)	UPS	5
**PF3D7_0826100**	MAL8P1.23	E3 ubiquitin-protein ligase, putative	UPS	0.2
**PF3D7_1023900**	PF10_0232	chromodomain-helicase-DNA-binding protein 1 homolog, putative (CHD1)	Pyrimidine metabolism	0.5
**PF3D7_1235700**	PFL1725w	ATP synthase subunit beta, mitochondrial	Oxidative phosphorylation	0.2
**PF3D7_1465900**	PF14_0627	40S ribosomal protein S3	Ribosome, translation	5.5
**PF3D7_0316800**	PFC0735w MAL3P6.30	40S ribosomal protein S15A, putative	Ribosome, translation	0.1
**Trophozoite (cAMP)**				
**PF3D7_0724300**	MAL7P1.130	3-demethylubiquinone-9 3-methyltransferase, putative	UPS	3.4
**PF3D7_1338100**	MAL13P1.190	26S proteasome regulatory subunit RPN3, putative (RPN3)	UPS	3.3
**PF3D7_0313100**	MAL3P4.5, PFC0550w	ubiquitin-protein ligase, putative (HRD3)	UPS	0.4
**PF3D7_1116000**	PF11_0168, RON4	rhoptry neck protein 4 (RON4)	Host cell invasion	0.1
**Schizont (cAMP)**				
**PF3D7_1458500**	PF14_0558	DN spindle assembly abnormal protein 4, putative (SAS4) A replication	DNA duplication	5.3
**PF3D7_1021800**	PF10_0212, PF10_0212a	schizont egress antigen-1 (SEA1)	DNA duplication	2.1
**PF3D7_0514600**	MAL5P1.146, PFE0730c	ribose 5-phosphate epimerase, putative	Coenzyme metabolic process	4.1
**PF3D7_1243700**	2277.t00420, MAL12P1.418, PFL2100w	ubiquitin-conjugating enzyme E2, putative	Acid-amino acid ligase	0.2
**PF3D7_1240600**	2277.t00392, MAL12P1.390, PFL1960w	proteasome regulatory protein, putative	UPS	4
**PF3D7_1311500**	PF13_0063	26S protease regulatory subunit 7, putative (RPT1)	UPS	0.4
**PF3D7_1243700**	2277.t00420, MAL12P1.418, PFL2100w	ubiquitin-conjugating enzyme E2, putative	UPS	0.2
**Trophozoite (MEL)**				
**PF3D7_0704600**	-	E3 ubiquitin ligase	UPS	3
**PF3D7_1402500**	-	40S ubiquitin proteasome	UPS	7
**PF3D7_1021800**	-	PfSEA1	host cell membrane	4
**PF3D7_1428800**	-	Transcription TFIID like	DNA-dependent transcription	0.1
**PF3D7_0322400**	-	eIF2	UPS	42

* Values above 1.0 represent up-regulated genes. (e.g., fold change = 5.0 means that the expression levels of these genes in treated parasites are 5 times higher compared to those in the controls), values below 1.0 represent down-regulated genes (e.g., fold change = 0.2 means that the expression levels pf these genes in treated parasites are 5 times lower compared to those in the controls).

The NIMA-related serine/threonine kinases NEK3 mRNA expression levels increased due to cAMP treatment. NEK3 belongs to the NEK family of proteins, which are important for DNA replication and which have been implicated as possible regulators of an atypical mitogen-activated protein kinase (MAPK) because both enzymes synergize with Pfmap-2 MAPK *in vitro* [62].

cAMP treatment of infected RBCs led to an increase in the proportion of schizonts; we found that an increase in the mRNA expression of genes involved in the cell cycle and DNA replication, such as RPA1 [63], SEA1, NDK and the myb2 transcription factor. The up-regulation of this set of genes partially explains how the cAMP-treated parasite maintains a large number of schizonts. RPA1 gene activity correlates with the timing of chromosomal DNA replication [63]. Antibodies against PfSEA1 decreased parasite replication and blocked schizont rupture [60], suggesting that this protein is important for merozoite egression. SAS-4 protein is exclusively expressed in the late S stage [64]. Myb proteins bind DNA in a sequence- specific manner and regulates the expression of genes involved in differentiation and growth control [65].

**Figure 6:**
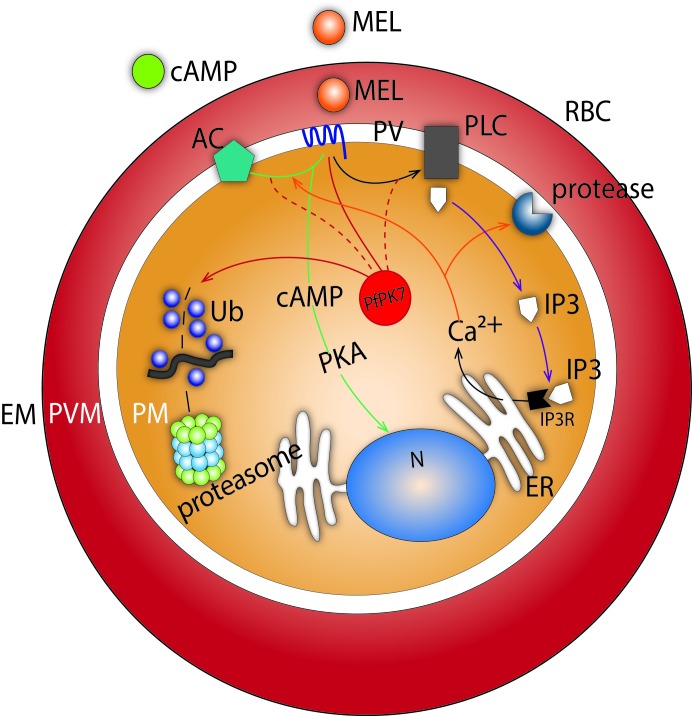
Melatonin and cAMP pathway modulation during the asexual cycle of *P. falciparum* development The results in Table 2 were used to identify the pathways presented here.

cAMP negatively modulates genes involved in the UPS and protein degradation, such as E2 ubiquitin, 26S proteasome, APC and falstatin. Likewise, in T and S stages, cAMP also regulates UPS genes. These data suggest that cAMP may modulate UPS genes independent of the activation of a MEL-mediated signaling cascade. Not only UPS genes but also genes involved in the cell cycle, cell invasion and egress appear to be modulated independent of the MEL cascade. Figure 6 shows a schematic of MEL and cAMP action during the *P. falciparum* asexual stage of development.

Our results suggest that genes involved in replication, release and merozoite invasion, such as CCR4, which is crucial for the regulation of proteins involved in egression and invasion events, may be excellent candidates for controlling parasite proliferation and for designing new antimalarial drugs.

## MATERIALS AND METHODS

### *Plasmodium falciparum* culture

*Plasmodium falciparum* parasite lines (3D7, *PfPK7*- and PfPK7C clones) were cultured in 25 cm^2^ flasks at 37°C and 5% hematocrit in RPMI 1640 medium supplemented with 10% human plasma, gassed with 90% N_2_, 5% O_2_ and 5% CO_2_. The synchronous parasite was obtained from R stage parasites, incubated for 10 minutes with 5 % sorbitol solution culture, and centrifuged for 5 minutes at 800 × *g* at room temperature. The supernatant was discarded, and the erythrocytes were washed twice with complete RMPI medium and added back to the normal culture as previously described[66]. For *PfPK7*- and PfPK7C parasites, the same medium was supplemented with 2.5 μg/mL blasticidin.

### Parasite synchronization

The *P. falciparum* 3D7 strain culture was highly synchronized as follows: the cultured parasites that had reached the R stage were centrifuged at ~300 × *g* for 5 minutes at room temperature. The medium was discarded, and parasite-infected RBCs were incubated with 5% sorbitol for 10 minutes. After treatment, the culture was centrifuged again, and the sorbitol was removed. The culture was washed twice with PBS, and RPMI medium was added [67]. The knockout strain PfPK7 was kindly provided by Professor Christian Doerig [68].

### Melatonin treatment

The cultured erythrocytes previously synchronized with parasites were transferred to a large flask, and fresh medium was added. MEL (final concentration, 100 nM) was immediately added to the synchronized parasites in culture at the T stage (30 hours after infection) and incubated for 5 hours [6] at 37°C. Every treatment was performed on two biological replicates.

### 6-BnZ-cAMP analog treatment

All experiments were performed using human erythrocytes. The culture of erythrocytes infected with *P. falciparum* was highly synchronized using sorbitol. Intraerythrocytic development cycle of *P. falciparum* was monitored by smear and Giemsa staining. Malaria parasites have a 48-hour asexual cycle inside RBCs; thus, the selected hours were representative of the 3 stages (R, T, and S). R, T, and S parasites at 6 hours, 34 hours and 44 hours, respectively, were used for cAMP assays. At each timepoint, 20 μM 6-Bnz-cAMP analog was added, and the cultures were incubated for 6 hours at 37°C. Every treatment was performed on two biological replicates.

### RNA extraction and library construction

Total RNA extraction was performed following the protocol of Ponts et al. [69], with some modifications. Parasites from highly synchronous cultures were harvested at 12, 30, 40 and 50 hours post-infection and after MEL and cAMP treatments. The cultures were centrifuged at 700 × *g* for 5 minutes, and then the supernatant was removed. Five volumes of room temperature TRIzol® LS (Invitrogen, Carlsbad, CA, USA) were added and mixed thoroughly. The samples were incubated at 37°C for 5 minutes to ensure the complete deproteinization of nucleic acids. On ice, 1 mL of chloroform was added to the samples, and then the samples were centrifuged at 12000 × *g* for 30 minutes at 4°C. The upper aqueous layer was transferred to a new tube, and 0.8 volume of pre-chilled isopropanol was added to precipitate the RNA. The tubes were centrifuged at 12000 × *g* for 30 minutes at 4°C. The supernatant was carefully aspirated off. The pellet was air-dried on ice for 5 minutes, and 30-100 μL of RNase-free non-DEPC-treated water was added. The isolated RNA was quantified using a NanoDrop ND- 1000 UV/Vis spectrophotometer, and RNA quality was determined using a Bioanalyzer apparatus (Agilent 2100 Bioanalyzer). Total RNA (6000 ng) from each sample was stabilized in RNAStable (Biomatrica) and sent to Scripps Research Institute for HiSeq Illumina sequencing. For each sample, approximately 0.5 μg of total RNA was depleted of ribosomal RNA using RiboMinus (Life Technologies). Ribo-depleted RNA was prepared for sequencing using an NEBNext Ultra RNA Library Prep Kit (New England Biolabs) for Illumina following the manufacturer's recommended protocols with 15 PCR cycles. Final libraries were size selected on 2% agarose gels to obtain products with library sizes between 280- 380 bases in length. The prepared libraries were loaded onto an Illumina HiSeq 2000 platform for single-end 100 base reads with 7 bases of the index read. The data were processed to generate Fastq files using CASAVA 1.8 and multiplexed based on index sequences.

### RNA-Seq data analysis

The reference genome and transcript information for *P. falciparum* 3D7 v9.3 were obtained from PlasmoDB (http://plasmodb.org/). First, FastQC (http://www.bioinformatics.babraham.ac.uk/projects/fastqc/) was used to analyze the quality of the reads. Next, reads were mapped to the reference genome using Tophat2 [70] (parameters: -no-coverage-search and -p 24). Samtools was used to select only those reads with a minimum mapping quality of 20 (-q 20). The gene expression profile for each sample was obtained using HTSeq-Count [71] with the parameter “intersection-strict mode”. The total read count for each samples was used in the subsequent analyses. Differential expression analyses following MEL treatment were performed using EdgeR [72]. A log-fold change greater than 1.5 or lower than −1.5, CPM ≥ 1, FDR < 0.05, and p-value < 0.05 were selected as filters for defining the sets of differentially expressed (up- or down-regulated) genes. Differential expression analyses following cAMP treatment were performed using EdgeR [72] and DeSeq [73]. A log-fold change greater than 2.0 or lower than −2.0, CPM ≥ 1, and p-value < 0.05 were selected as filters for defining the sets of differentially expressed (up- or down-regulated) genes.

### Functional enrichment analysis

DAVID Bioinformatics Resources v6.7 was used to perform functional enrichment analysis [30, 74]. The web- accessible DAVID program provides a comprehensive set of functional annotation tools to understand the biological meaning behind large lists of genes. The functional categorization is considered significant when the p-value is less than 0.05. To gain more information regarding our set of up- and down-regulated genes, GO terms were assigned manually to those genes using PlasmoDB annotation as a reference. KEGG pathway mapping is used to map molecular datasets, especially large-scale datasets in genomics, transcriptomics, proteomics, and metabolomics, to the KEGG pathway maps for biological interpretation of higher-level systemic functions. Therefore, we used KEGG Mapper (http://www.genome.jp/kegg/tool/map_pathway1.html) against the *P. falciparum* 3D7 database to identify those pathways that were modulated by cAMP treatment.

## SUPPLEMENTARY FILES


