# Supplementary material for: Signaling transcript profile of the asexual intraerythrocytic development cycle of Plasmodium falciparum induced by melatonin and cAMP
Source: Genes Cancer. 2016 Sep;7(9-10):323–39. doi: 10.18632/genesandcancer.118 (PMC5115173; doi:10.18632/genesandcancer.118)
Supplement: Supplementary file 1 [file ganc-07-323-s001.pdf]

Signaling transcript profile of the asexual intraerythrocytic development cycle of *Plasmodium falciparum* induced by melatonin and cAMP

Supplementary Files

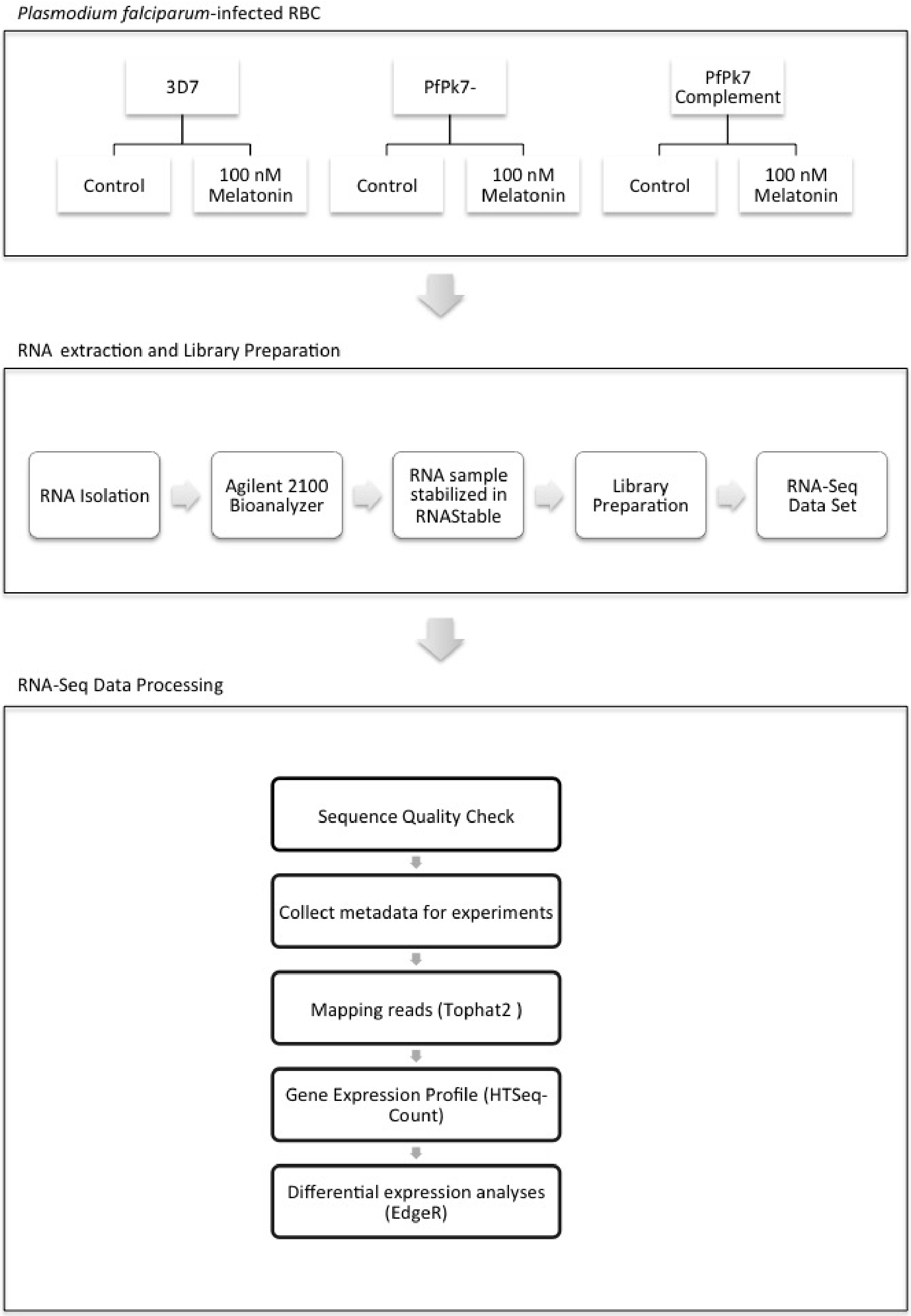

Supplementary Figure 1 – Experimental and computational workflow. Schematic overview of the methodology and data processing used to analyze the Melatonin RNA-Seq datasets

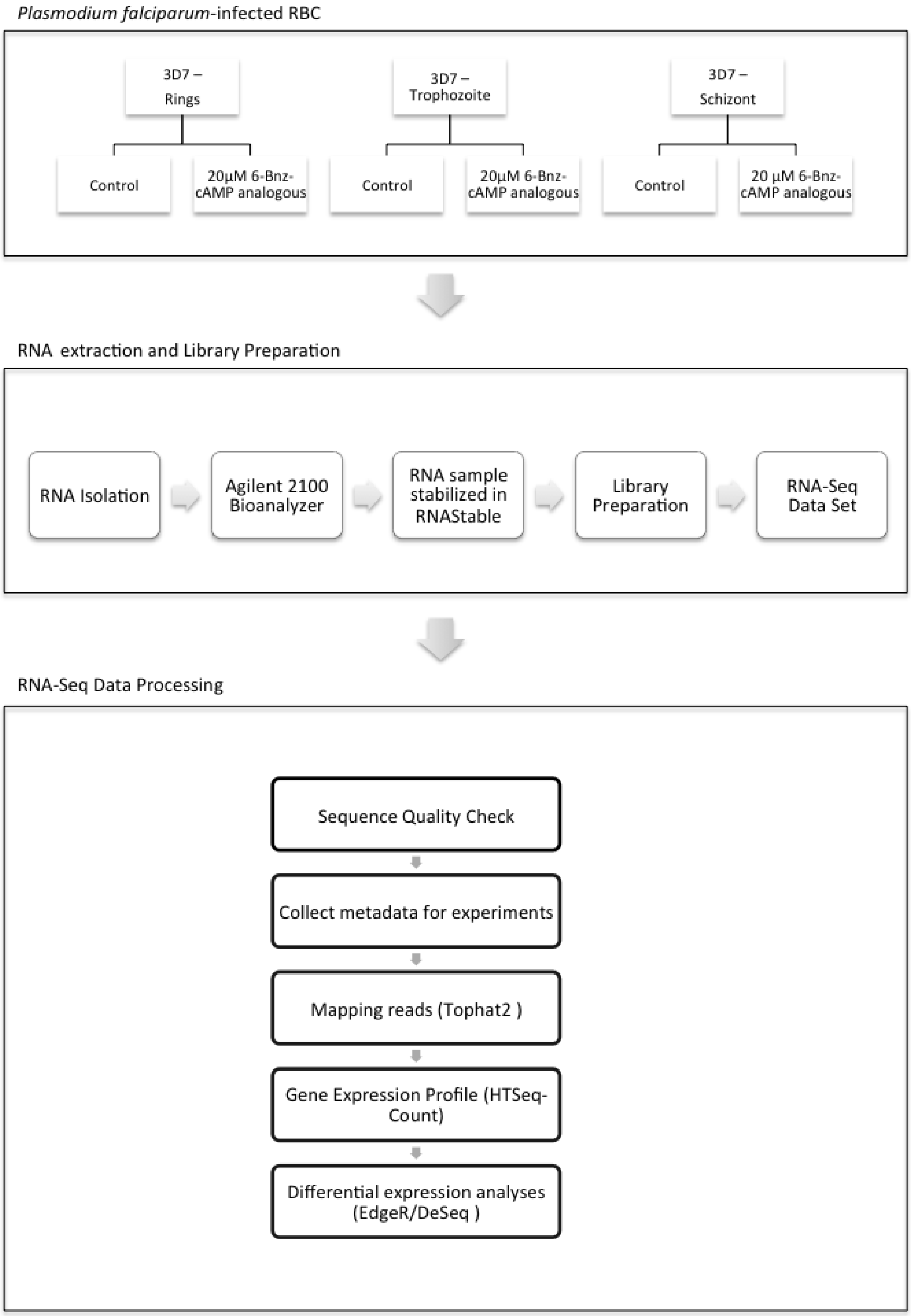

Supplementary Figure 2– Experimental and computational workflow. Schematic overview of the methodology and data processing used to analyze the 6-BnZ-cAMP analogous RNA-Seq datasets

Supplementary Table 1 - Summary of sequences (reads) generated and aligned (mapped) against the reference genome.

Sample	Total of reads	Total of mapped reads	Percentage
**3D7_Control_5H.sample1**	16822987	12254971	72.8
**3D7_Control_5H.sample2**	17539049	12544741	71.5
**3D7_Treated_5H.sample1**	12849142	9642937	75.0
**3D7_Treated_5H.sample2**	17515243	13429260	76.7
**Pk7_Comp_Control_5H.sample1**	13716973	9976511	72.7
**Pk7_Comp_Control_5H.sample2**	16308397	11598991	71.1
**Pk7_Comp_Treated_5H.sample1**	13925235	10411161	74.8
**Pk7_Comp_Treated_5H.sample2**	13135961	9697431	73.8
**Pk7_Control_5H.sample1**	13784591	10046120	72.9
**Pk7_Control_5H.sample2**	5955881	4528655	76.0
**Pk7_Treated_5H.sample1**	12662506	9249944	73.0
**Pk7_Treated_5H.sample2**	12961939	8534736	65.8

Supplementary Table 2 - FastQC quantification. There is a quantification for each of all 12 samples.

a) 3D7 Control 5H sample1

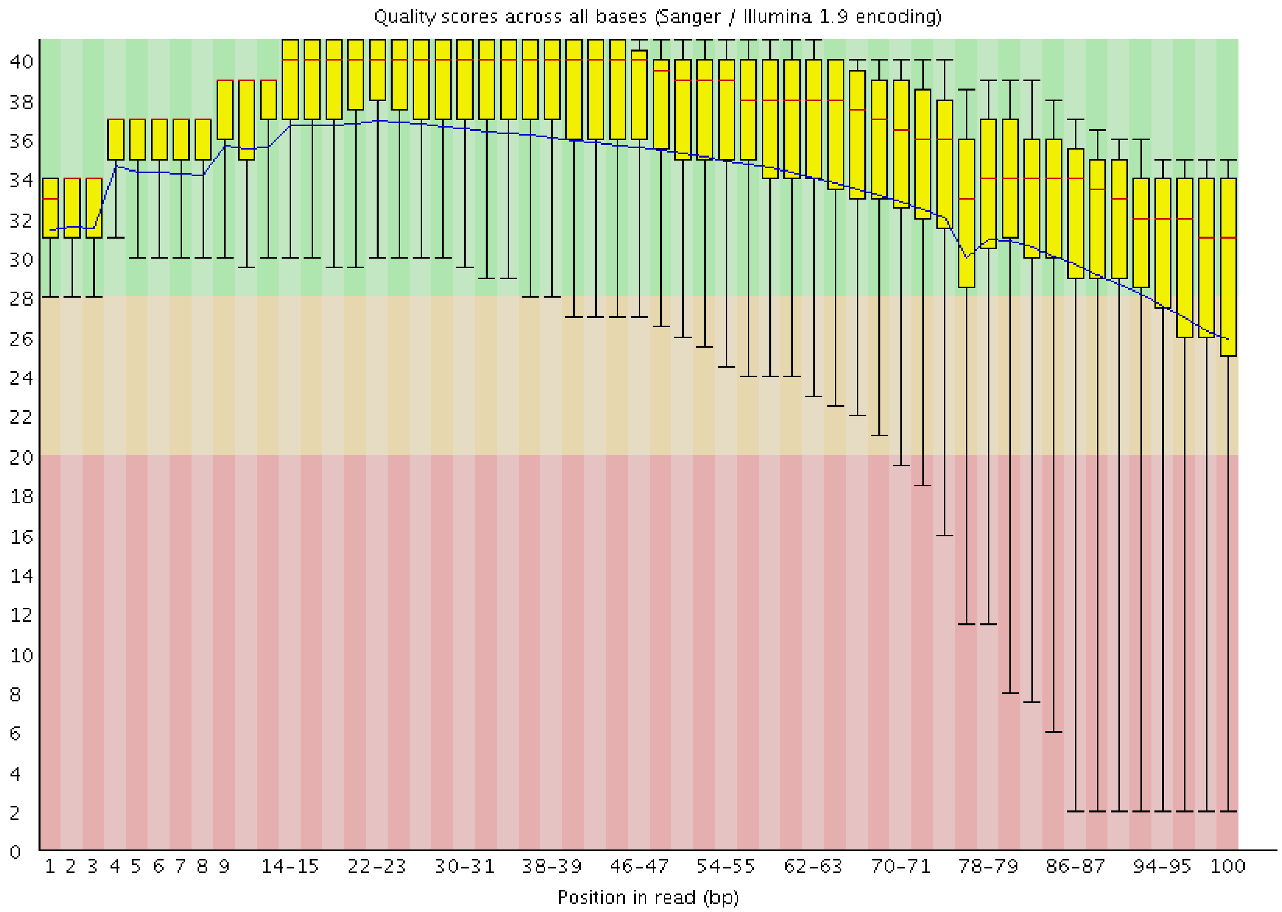

b) 3D7 Control 5H sample2

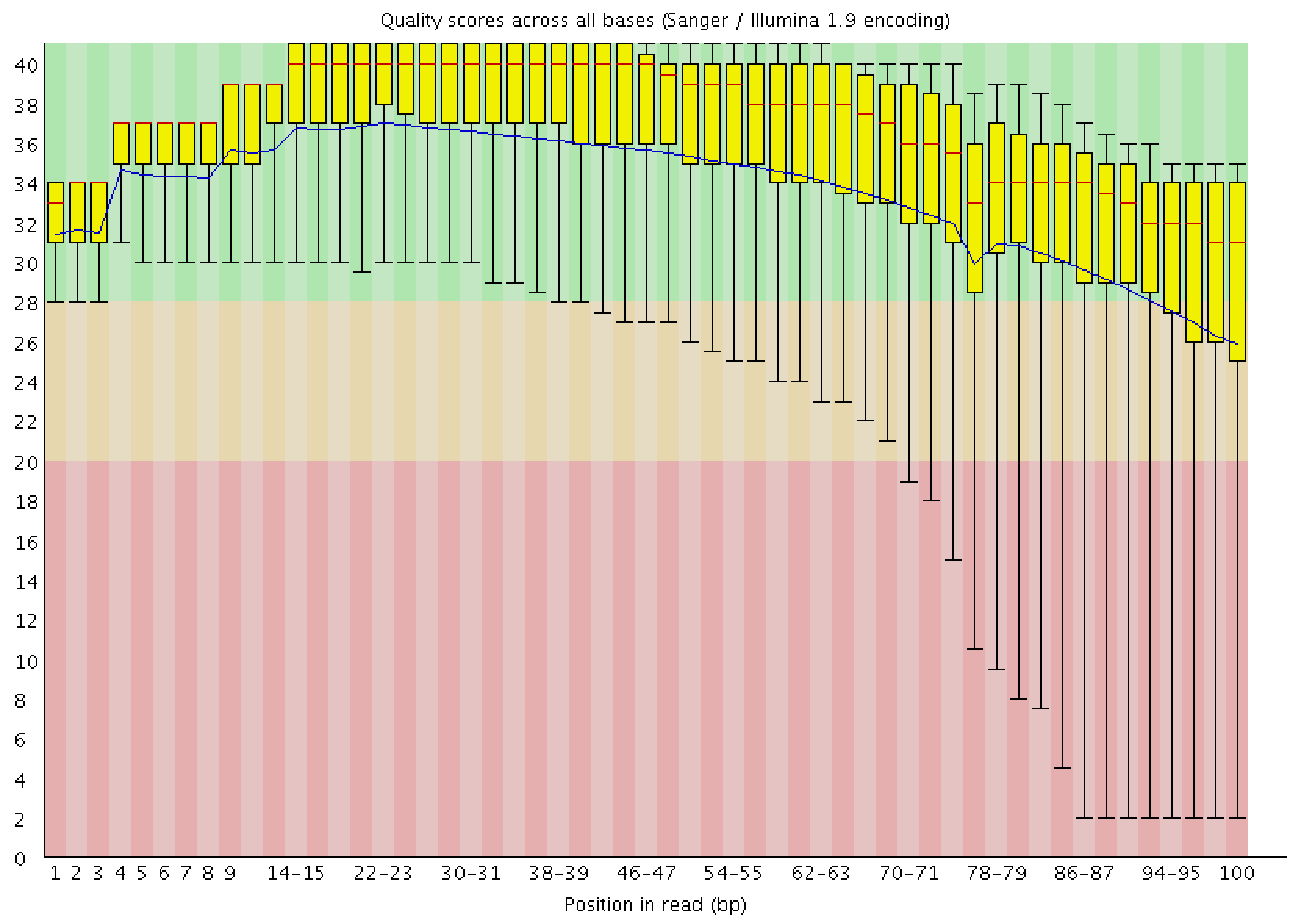

c) 3D7 Treated 5H sample1

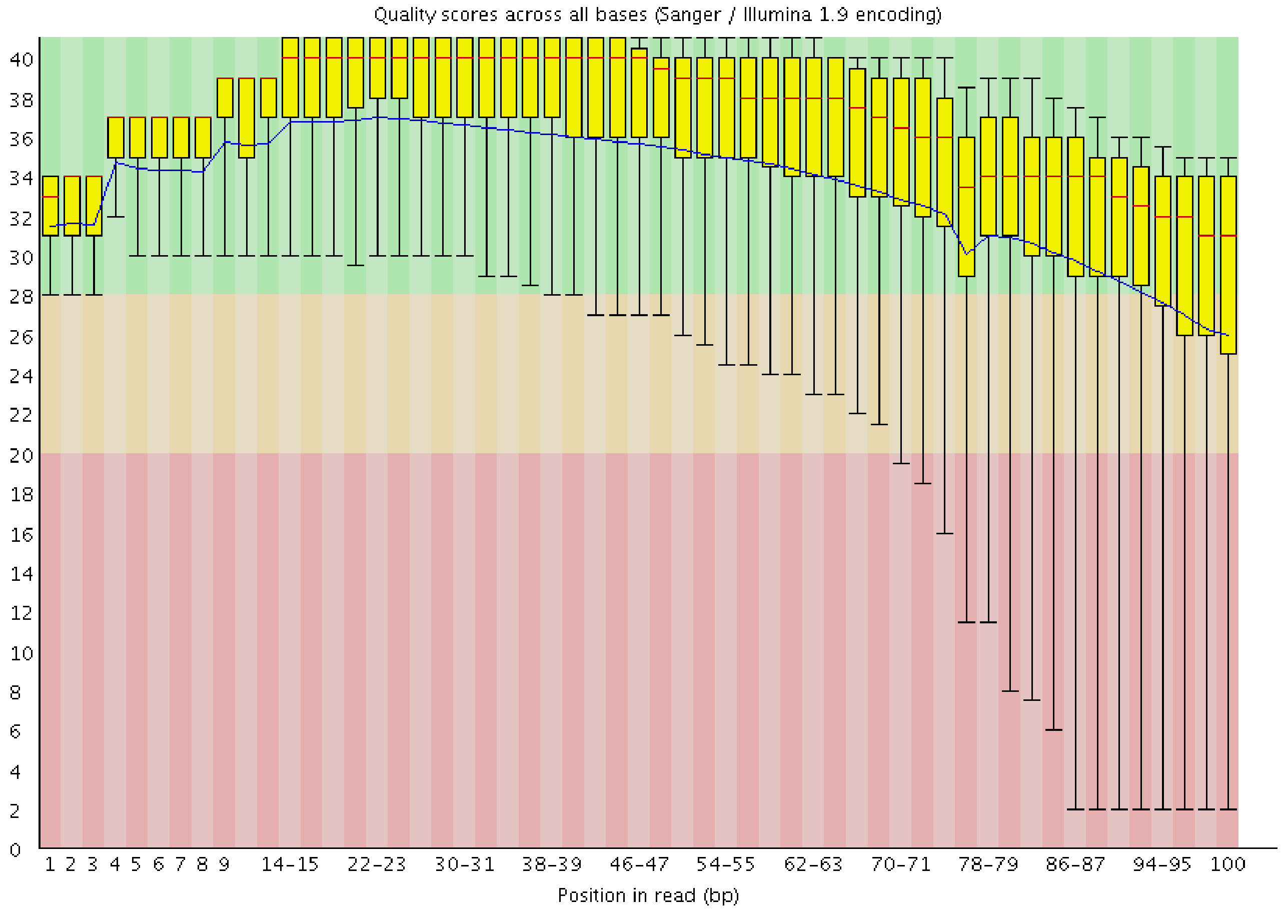

d) 3D7 Treated 5H sample2

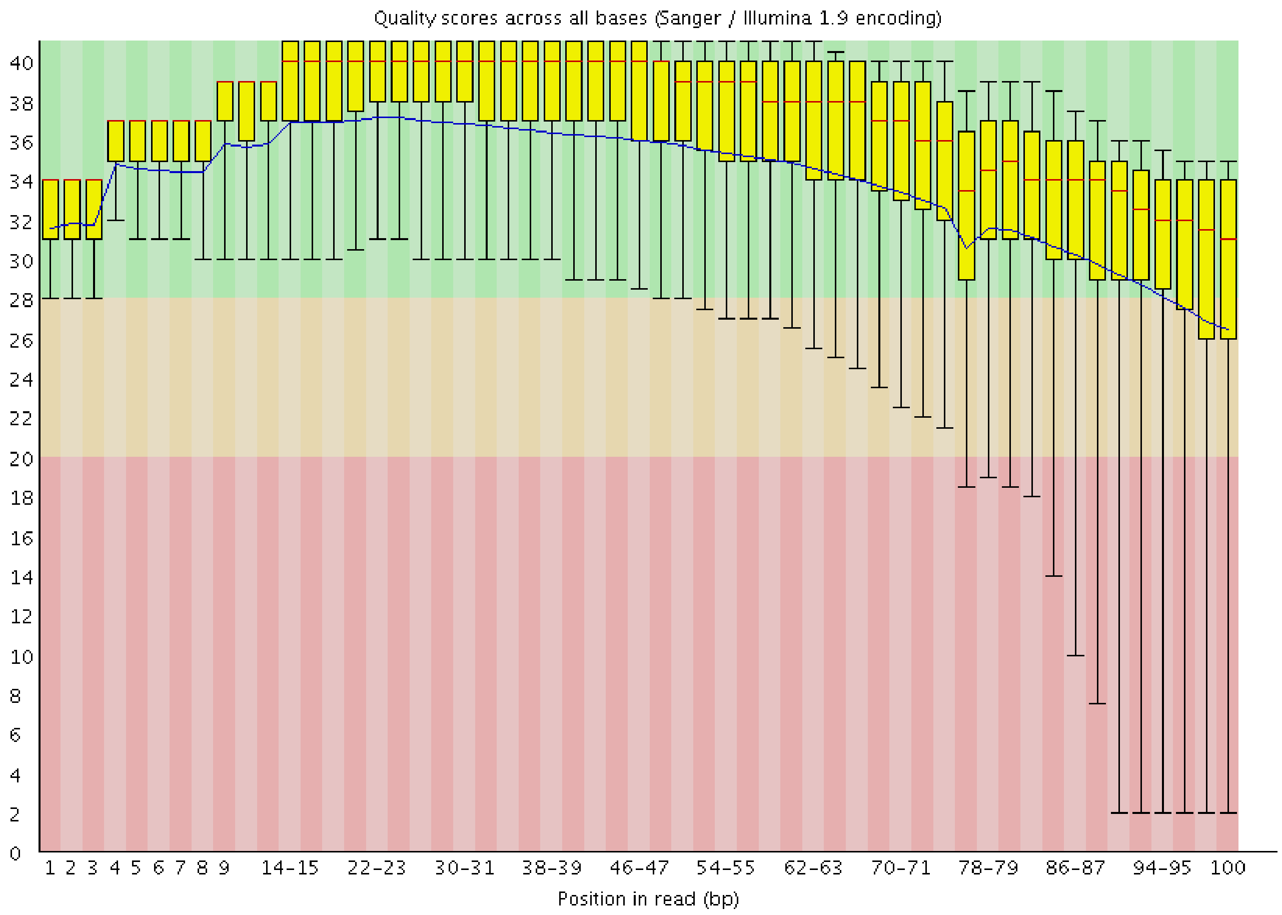

e) Pk7 Comp Control 5H sample1

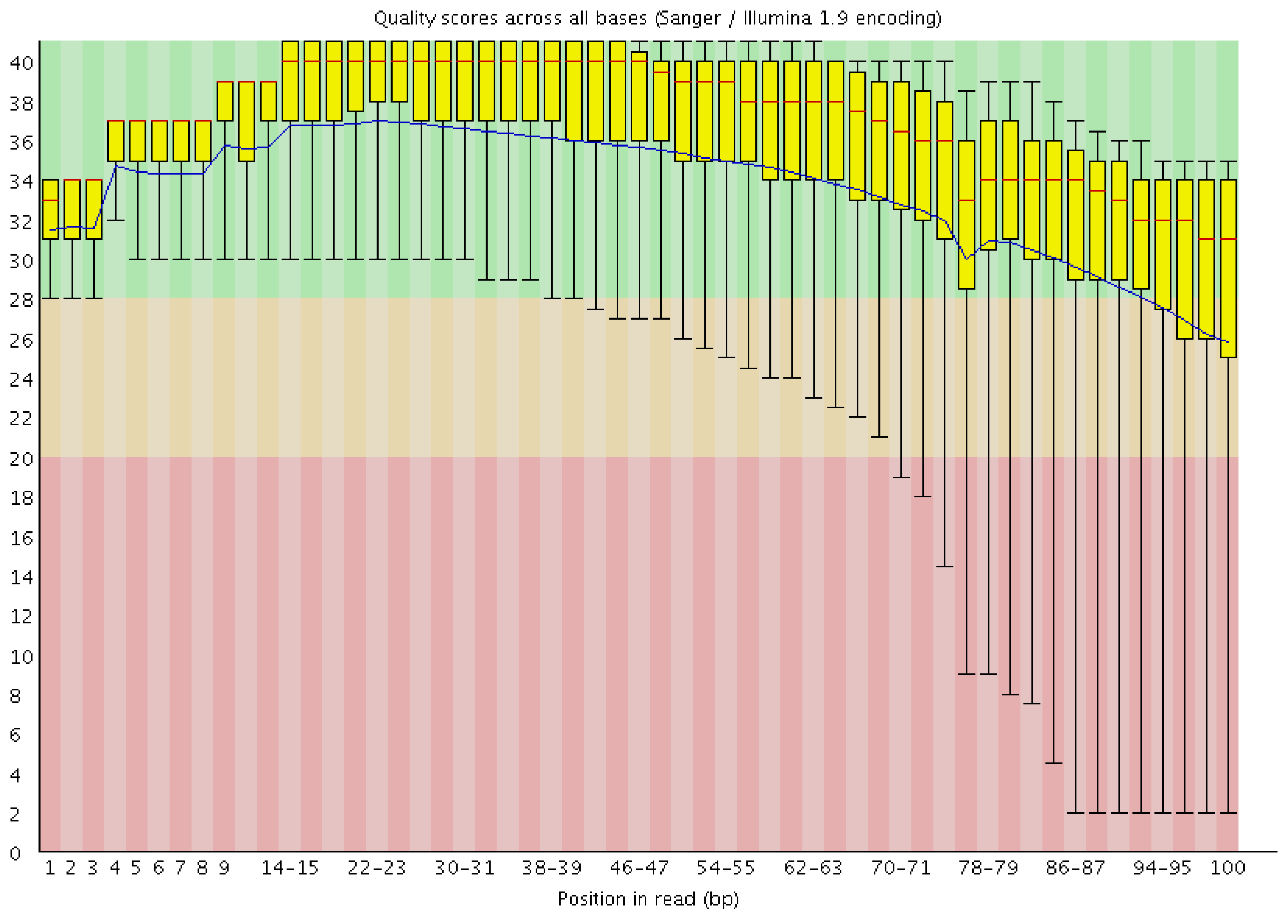

f) Pk7 Comp Control 5H sample2

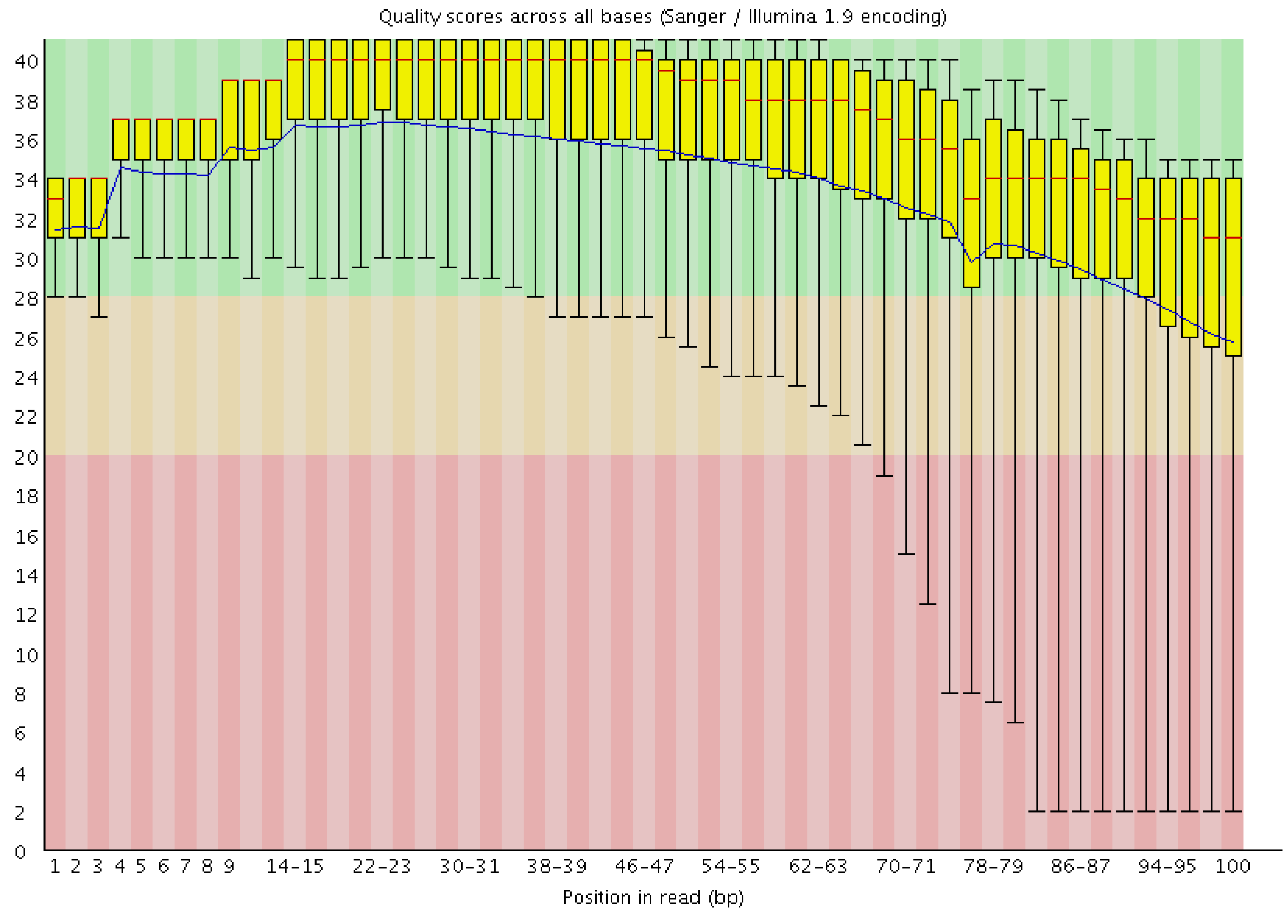

g) Pk7 Comp Treated 5H sample1

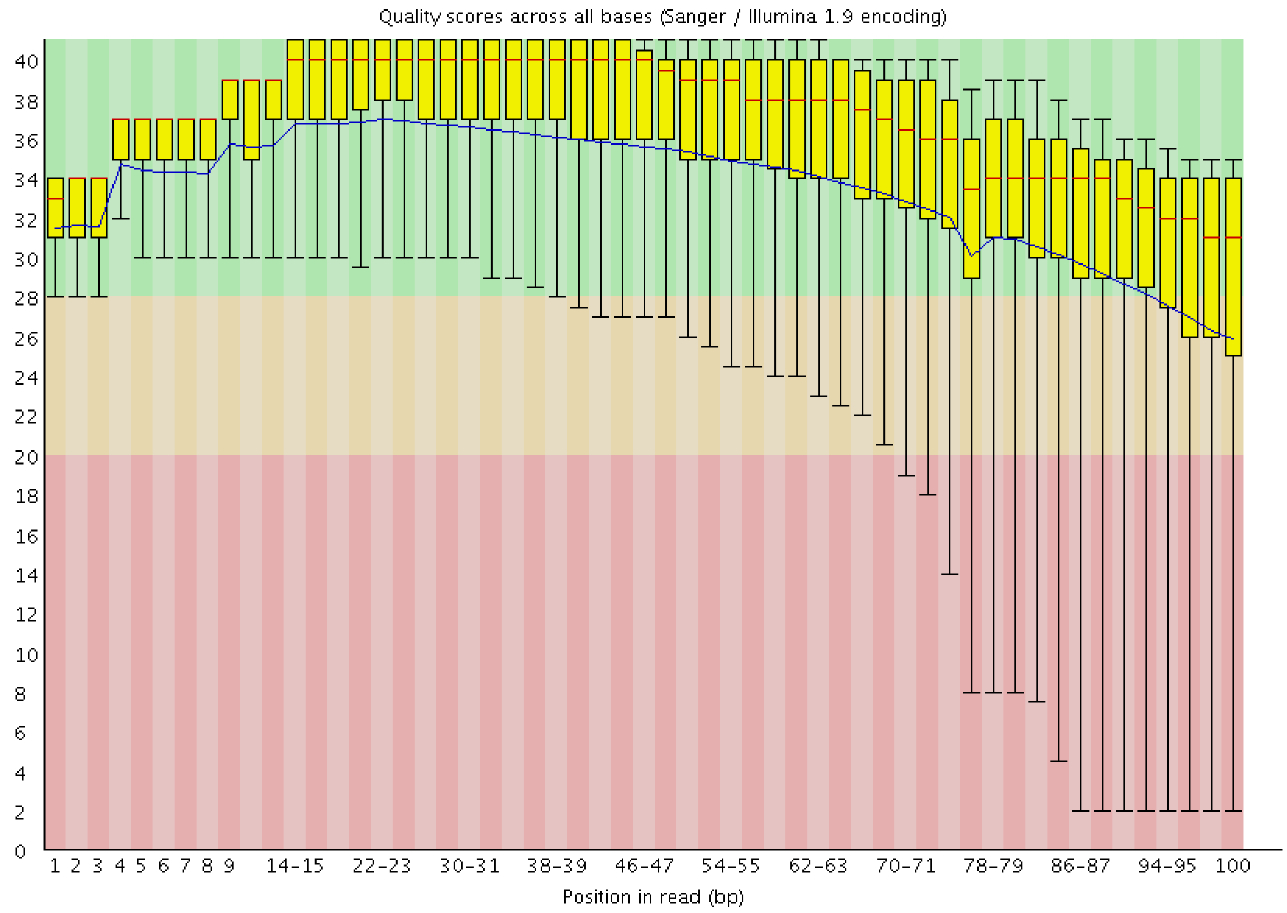

h) Pk7 Comp Treated 5H sample2

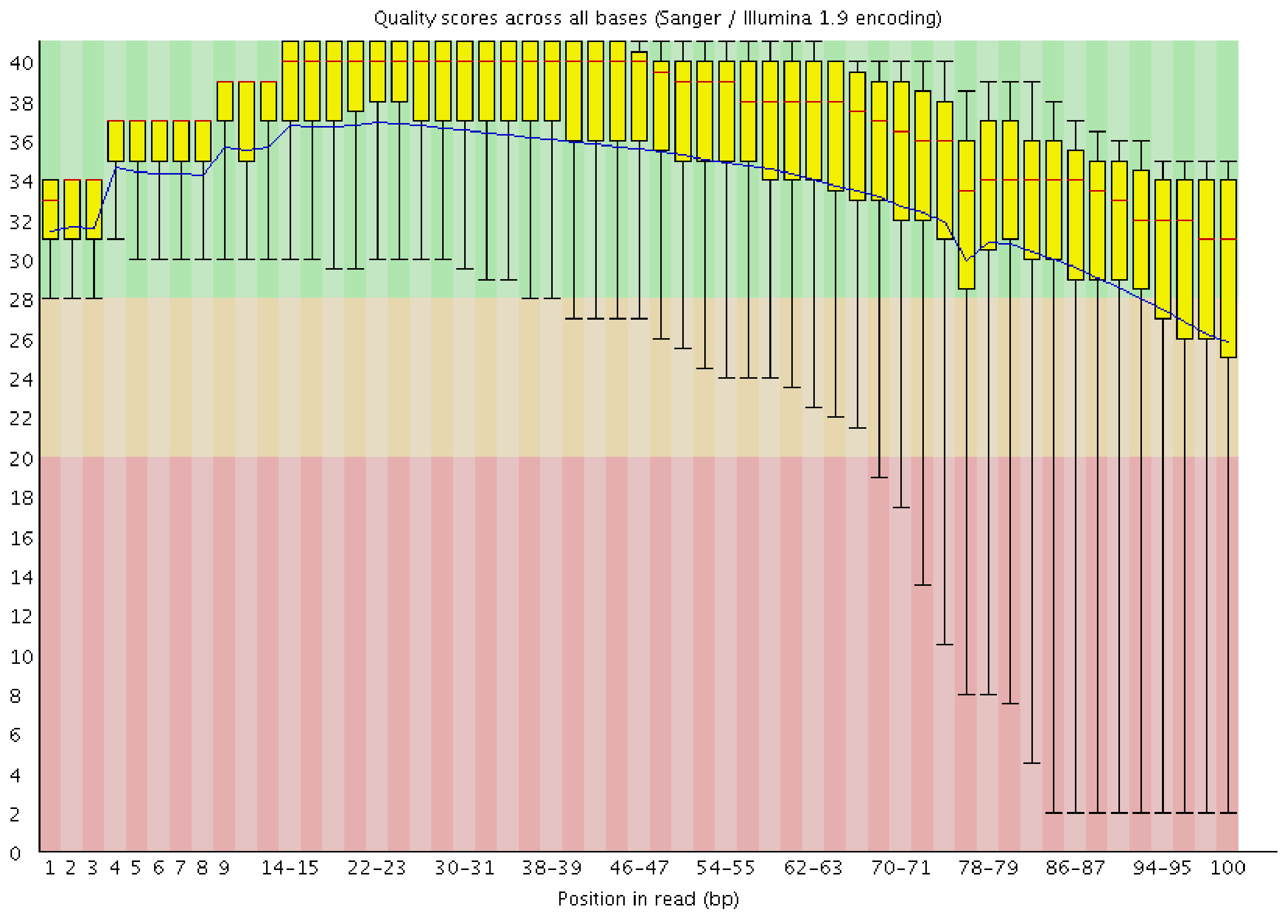

i) Pk7 Control 5H sample1

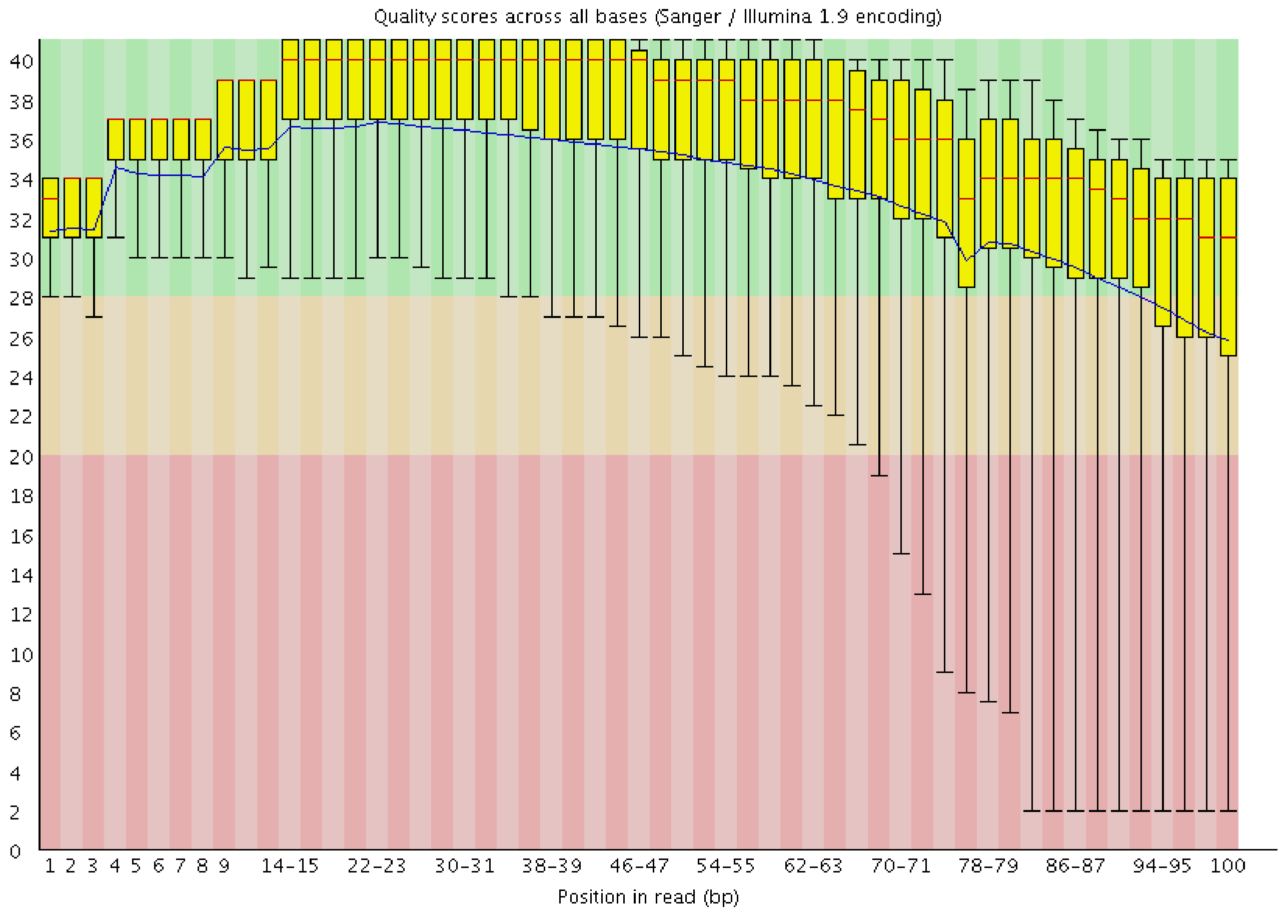

j) Pk7 Control 5H sample2

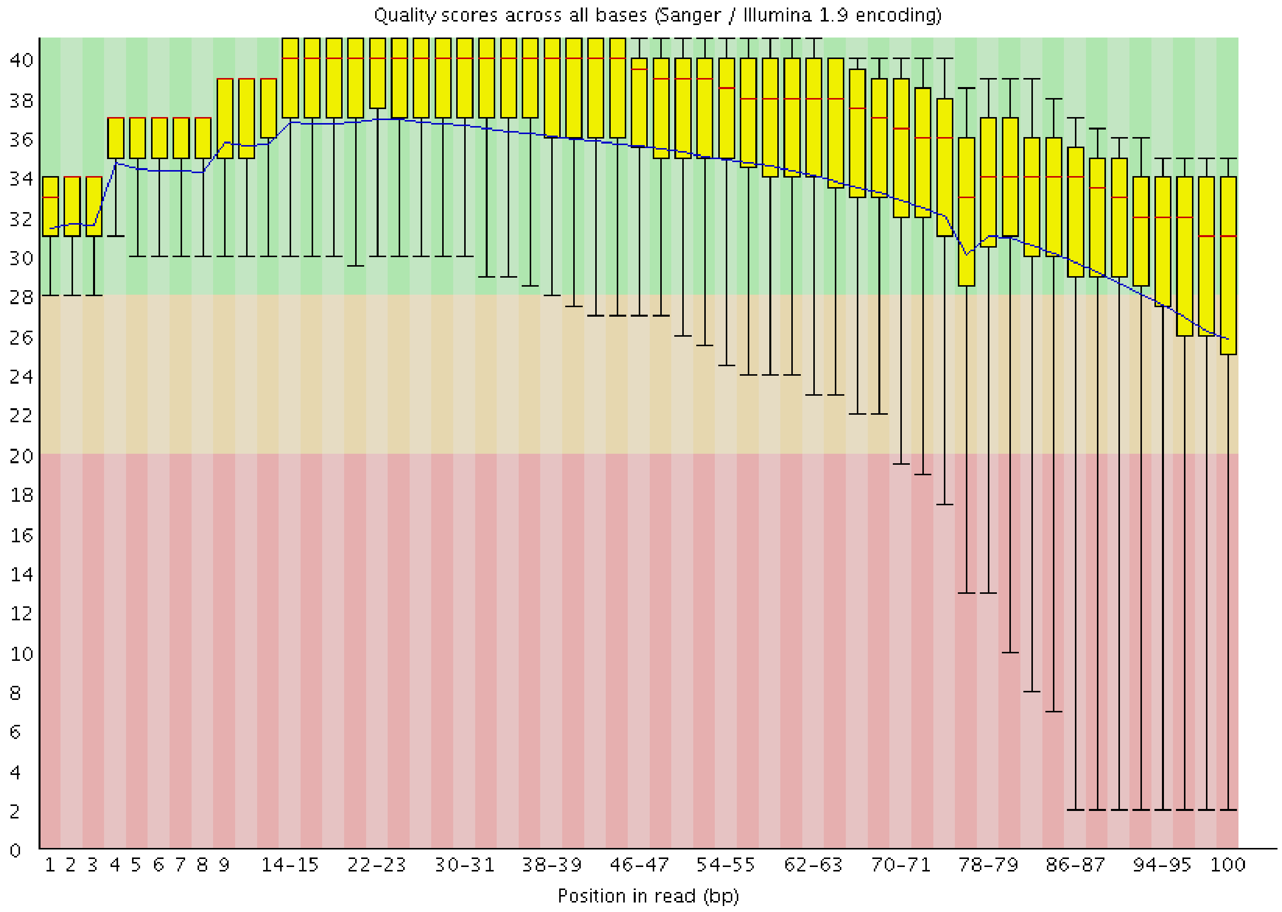

k) Pk7 Treated 5H sample1

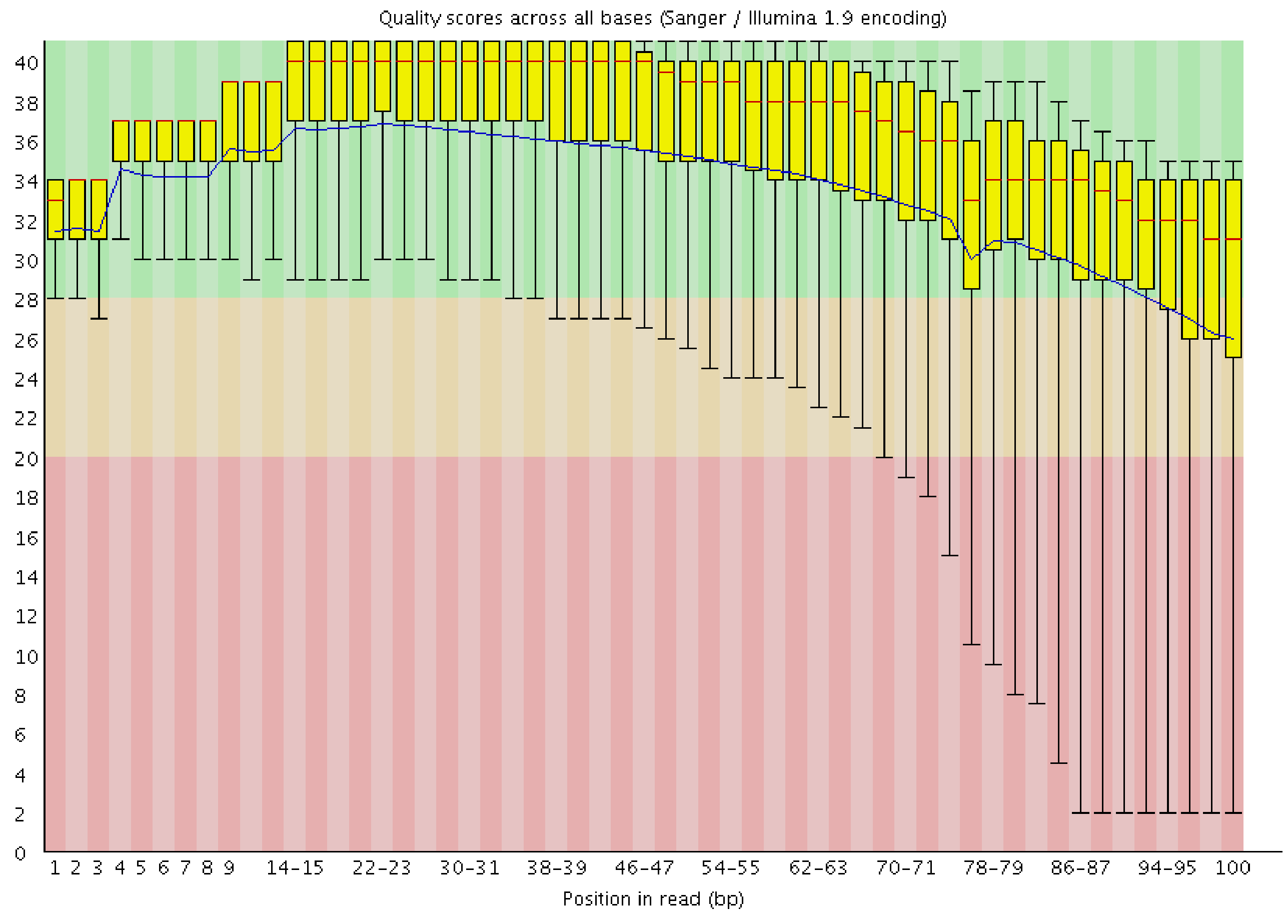

l) Pk7 Treated 5H sample2

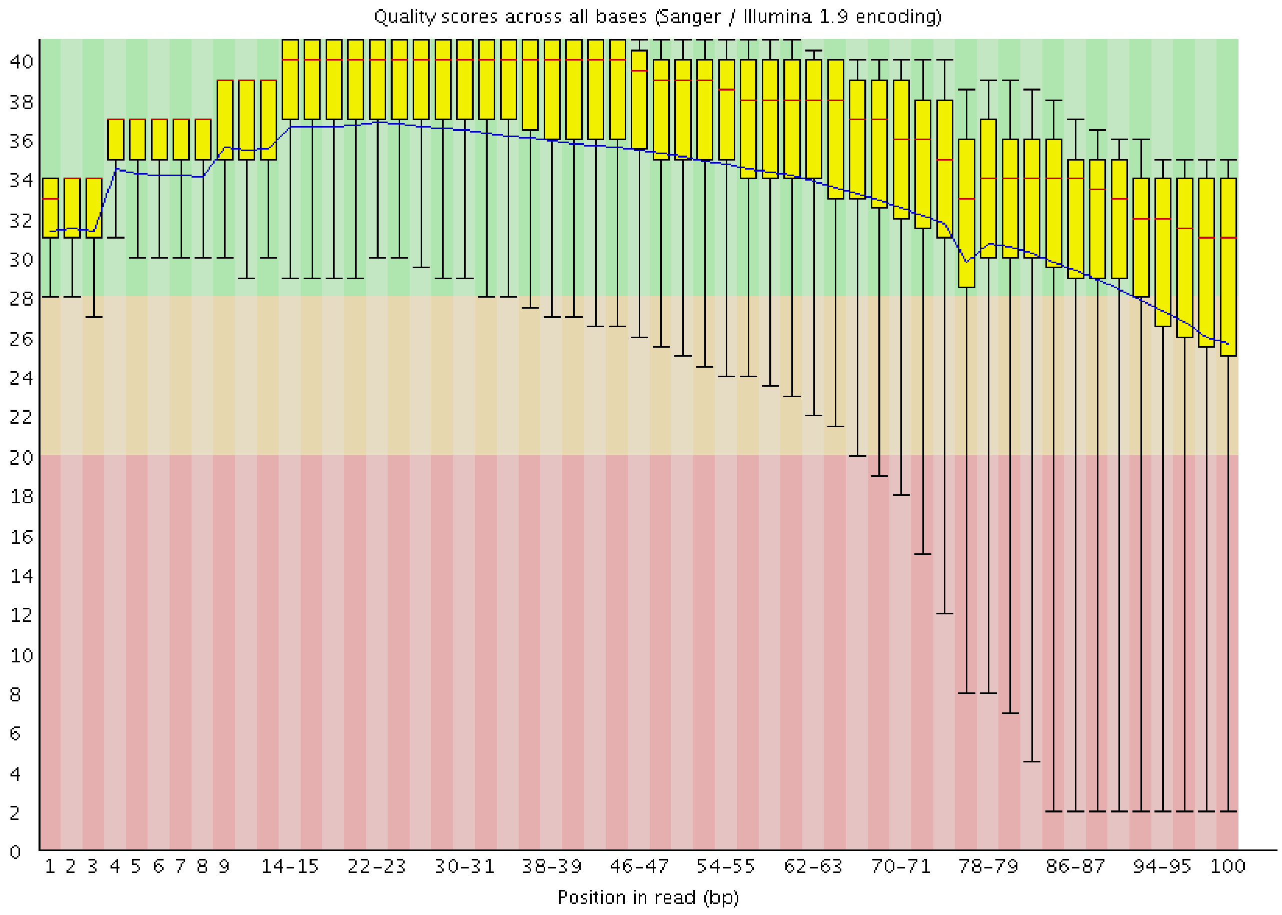

Supplementary Table 3 - Differentially expressed genes in 3D7 strain treated for 5h with melatonin

ID	Annotation	Fold Change (Log)	CPM (Log)
PF3D7_0811400	conserved protein, unknown function	5,704653526	7,291648204
PF3D7_0728000	eukaryotic translation initiation factor 2 alpha subunit, putative	5,703661315	7,289256335
PF3D7_0812100	conserved Plasmodium protein, unknown function	5,700067346	7,288061086
PF3D7_0322400	regulator of initiation factor 2 (eIF2)	5,389469076	7,094507153
PF3D7_0724700	conserved Plasmodium protein, unknown function	4,150743332	8,034654281
PF3D7_0206000	DNA repair endonuclease, putative	3,636497855	7,663795636
PF3D7_1239800	conserved Plasmodium protein, unknown function	3,562445557	7,620030462
PF3D7_1315400	zinc finger (CCCH type) protein, putative	3,370613975	8,144310244
PF3D7_1220900	heterochromatin protein 1 (HP1)	3,104161333	8,386412654
PF3D7_0212400	conserved Plasmodium membrane protein, unknown function	2,946122622	7,847161363
PF3D7_0910200	conserved Plasmodium protein, unknown function	2,929829329	8,866719904
PF3D7_1332800	translation initiation factor 6, putative	2,871953416	8,207242393
PF3D7_1006800	RNA-binding protein, putative	2,840083394	10,35079378
PF3D7_1402500	ubiquitin-40S ribosomal protein S27a, putative	2,80553105	8,495097751
PF3D7_0402400	Plasmodium exported protein, unknown function (GEXP18)	2,667829062	8,390337848
PF3D7_0922600	glutamine synthetase, putative	2,625676573	8,355495894
PF3D7_0103400	zinc-carboxypeptidase, putative	2,548179872	8,55429054
PF3D7_0504800	conserved Plasmodium protein, unknown function	2,517510701	8,279528091
PF3D7_1303800	conserved Plasmodium protein, unknown function	2,388542882	9,376130268
PF3D7_1252800	Plasmodium exported protein (PHISTb), unknown function	2,274737933	8,931044419
PF3D7_0730900	Plasmodium exported protein, unknown function	2,263718505	8,340519543
PF3D7_0716300	conserved Plasmodium protein, unknown function	2,251516669	10,62964831
PF3D7_1113100	protein tyrosine phosphatase (PRL)	2,244981626	8,559885358

PF3D7_1033100	S-adenosylmethionine decarboxylase/ornithine decarboxylase (AdoMetDC/ODC)	2,22190812	10,78771351
PF3D7_1346300	DNA/RNA-binding protein Alba 2 (ALBA2)	2,133578339	10,28916836
PF3D7_1021800	schizont egress antigen-1 (SEA1)	2,035484007	8,753278353
PF3D7_0811300	CCR4-associated factor 1 (CAF1)	1,901319497	9,248076769
mal_rna_11:rRNA	unspecified product (SSUA)	1,816022676	10,04841153
PF3D7_0108700	secreted ookinete protein, putative (PSOP24)	1,766201863	9,625653191
PF3D7_0704600	ubiquitin transferase, putative	1,579692885	10,59745899
mal_rna_14:rRNA	unspecified product (LSUA)	1,543387291	12,75104471
PF3D7_1428000	conserved Plasmodium membrane protein, unknown function	-2,140792487	8,732296876
PF3D7_0202900	zinc finger protein, putative	-2,73639879	7,839630931
PF3D7_0809700	RuvB-like helicase 1 (RUVB1)	-2,793574463	7,580582613
PF3D7_1428800	transcription initiation TFIID-like, putative	-3,242308856	8,487137583
PF3D7_1430000	conserved Plasmodium protein, unknown function	-5,12356924	6,72415321
PF3D7_1351600	glycerol kinase (GK)	-5,310941131	6,818530382
PF3D7_1433600	microsomal signal peptidase protein, putative (SPC1)	-5,876914829	7,20659032

Supplementary Table 4 -Differentially expressed genes in trophozoite stage treated with cAMP

Gene ID	Gene Symbol	Product Description	Transcript Length	Genomic Location	logFC	logCPM	LR	PValue	FDR	FC	KEGG
PF3D7_1116600	PF11_0173	oligosaccharyl transferase STT3 subunit, putative	2571	Pf3D7_11_v3: 626,709 - 629,432 (-)	2.811	6.707	6.690	0.010	0.851	7.0	K07151
PF3D7_0317300	MAL3P6.11	conserved Plasmodium protein, unknown function	10185	Pf3D7_03_v3: 699,544 - 709,728 (-)	2.624	6.618	5.485	0.019	0.854	6.2	
PF3D7_1024800	PF10_0242	conserved Plasmodium protein, unknown function	4395	Pf3D7_10_v3: 1,034,685 - 1,039,079 (+)	2.624	6.623	5.485	0.019	0.854	6.2	
PF3D7_0722400	MAL7P1.122	GTP-binding protein, putative	1182	Pf3D7_07_v3: 954,784 - 956,343 (+)	2.600	6.622	4.311	0.038	0.893	6.1	K06942
PF3D7_1108500	PF11_0097	succinyl-CoA synthetase alpha subunit, putative	984	Pf3D7_11_v3: 367,316 - 368,823 (+)	2.538	7.083	8.625	0.003	0.703	5.8	K01899
PF3D7_1237600	MAL12P1.362, PFL1820w	rRNA processing WD-repeat protein, putative	1509	Pf3D7_12_v3: 1,568,605 - 1,570,569 (+)	2.530	7.089	7.597	0.006	0.851	5.8	
PF3D7_0707800	MAL7P1.23	RAP protein, putative	3552	Pf3D7_07_v3: 362,005 - 365,779 (+)	2.410	6.525	4.316	0.038	0.893	5.3	
PF3D7_1238800	MAL12P1.374, PFL1880w	acyl-CoA synthetase (ACS11)	2379	Pf3D7_12_v3: 1,610,363 - 1,612,741 (+)	2.410	6.525	4.316	0.038	0.893	5.3	K01897
PF3D7_1239000	MAL12P1.376, PFL1890c	HD superfamily phosphohydrolase protein	2469	Pf3D7_12_v3: 1,621,237 - 1,623,705 (-)	2.410	6.525	4.316	0.038	0.893	5.3	
PF3D7_1209300	PFL0465c	zinc finger transcription factor, putative (KROX1)	4386	Pf3D7_12_v3: 428,495 - 432,880 (-)	2.410	6.531	4.316	0.038	0.893	5.3	
PF3D7_1328900	PF13_0151	conserved Plasmodium protein, unknown function	1194	Pf3D7_13_v3: 1,215,925 - 1,217,118 (-)	2.410	6.530	4.316	0.038	0.893	5.3	
PF3D7_0708500	PF07_0030	heat shock protein 86 family protein	2739	Pf3D7_07_v3: 385,583 - 388,321 (-)	2.291	6.941	5.743	0.017	0.851	4.9	
PF3D7_0913500	PFI0660c	protease, putative	1299	Pf3D7_09_v3: 582,340 - 583,968 (-)	2.281	6.946	5.942	0.015	0.851	4.9	
PF3D7_1118800	PF11_0195	conserved Plasmodium protein, unknown function	1176	Pf3D7_11_v3: 714,117 - 716,287 (-)	2.182	7.757	11.690	0.001	0.352	4.5	K05757

PF3D7_0518500	MAL5P1.185, PFE0925c	ATP-dependent RNA helicase DDX23, putative (DDX23)	3372	Pf3D7_05_v3: 763,532 - 766,903 (-)	2.134	6.872	4.434	0.035	0.893	4.4	K12858
PF3D7_0501700	MAL5P1.18, PFE0085c	anaphase promoting complex subunit, putative	2382	Pf3D7_05_v3: 86,820 - 89,201 (-)	2.134	6.872	4.436	0.035	0.893	4.4	
PF3D7_1406400	PF14_0061	PPR repeat protein (PPR)	1827	Pf3D7_14_v3: 230,864 - 232,690 (+)	2.133	6.871	5.207	0.022	0.854	4.4	K09560
PF3D7_1440100	PF14_0380	conserved Plasmodium protein, unknown function	3045	Pf3D7_14_v3: 1,633,211 - 1,636,255 (-)	2.133	6.870	5.312	0.021	0.854	4.4	
PF3D7_1323900	PF13_0136	conserved Plasmodium protein, unknown function	1131	Pf3D7_13_v3: 989,468 - 990,598 (+)	2.123	7.207	7.441	0.006	0.851	4.4	K15262
PF3D7_1342600	PF13_0233	myosin A (MyoA)	2457	Pf3D7_13_v3: 1,673,699 - 1,676,539 (+)	1.967	6.791	4.280	0.039	0.893	3.9	
PF3D7_0305400	PFC0241w	conserved Plasmodium protein, unknown function	438	Pf3D7_03_v3: 260,332 - 261,085 (+)	1.938	7.385	7.393	0.007	0.851	3.8	
PF3D7_0724300	MAL7P1.130	3-demethylubiquinone-9 3-methyltransferase, putative	1005	Pf3D7_07_v3: 1,021,508 - 1,022,630 (+)	1.752	7.011	4.212	0.040	0.893	3.4	K00568 2
PF3D7_1428900	PF14_0268	conserved Plasmodium protein, unknown function	4338	Pf3D7_14_v3: 1,134,854 - 1,139,191 (-)	1.748	7.018	4.026	0.045	0.893	3.4	
PF3D7_0206500	PFB0285c	conserved Plasmodium protein, unknown function	4311	Pf3D7_02_v3: 263,037 - 267,347 (-)	1.737	7.263	5.654	0.017	0.851	3.3	
PF3D7_1338100	MAL13P1.190	26S proteasome regulatory subunit RPN3, putative (RPN3)	1512	Pf3D7_13_v3: 1,542,080 - 1,543,981 (+)	1.737	7.262	5.654	0.017	0.851	3.3	
PF3D7_0103800	MAL1P1.24, PFA0190c	actin-related protein (ARP1)	1143	Pf3D7_01_v3: 169,079 - 171,273 (-)	1.635	7.425	5.942	0.015	0.851	3.1	K16575
PF3D7_0627800	MAL6P1.150, PFF1350c	acetyl-CoA synthetase, putative (ACS)	2994	Pf3D7_06_v3: 1,114,544 - 1,117,537 (-)	1.537	7.376	5.079	0.024	0.870	2.9	K01897
PF3D7_0404700	MAL4P1.45, PFD0225c, PFD0230c	dipeptidyl aminopeptidase 3 (DPAP3)	2820	Pf3D7_04_v3: 260,454 - 263,418 (-)	1.432	7.321	4.253	0.039	0.893	2.7	
PF3D7_1368200	MAL13P1.344	RNAse L inhibitor protein, putative	1860	Pf3D7_13_v3: 2,713,463 - 2,715,436 (-)	1.428	7.672	5.669	0.017	0.851	2.7	K06174
PF3D7_0810500	MAL8P1.108	protein phosphatase, putative	912	Pf3D7_08_v3:	1.384	7.895	5.753	0.016	0.851	2.6	K17500

				534,469 - 536,619 (-)							
PF3D7_1428200	PF14_0260	metabolite/drug transporter, putative	2430	Pf3D7_14_v3: 1,101,958 - 1,104,387 (+)	1.352	7.615	4.787	0.029	0.893	2.6	
PF3D7_1410500	PF14_0103	conserved Plasmodium protein, unknown function	402	Pf3D7_14_v3: 423,751 - 424,250 (-)	1.344	8.018	6.003	0.014	0.851	2.5	
PF3D7_0703500	MAL7P1.12	erythrocyte membrane-associated antigen	6900	Pf3D7_07_v3: 135,606 - 142,505 (+)	1.334	7.744	4.747	0.029	0.893	2.5	
PF3D7_0724700	MAL7P1.133	conserved Plasmodium protein, unknown function	10470	Pf3D7_07_v3: 1,034,969 - 1,046,109 (-)	1.323	7.748	5.163	0.023	0.854	2.5	
PF3D7_1211900	MAL12P1.118, PFL0590c	non-SERCA-type Ca2+ -transporting P-ATPase (ATP4)	3795	Pf3D7_12_v3: 529,005 - 532,799 (-)	1.298	7.872	5.637	0.018	0.851	2.5	
PF3D7_1411900	PF14_0117	p1/s1 nuclease, putative	984	Pf3D7_14_v3: 480,431 - 481,414 (+)	1.287	8.628	8.756	0.003	0.703	2.4	
PF3D7_1029600	PF10_0289	adenosine deaminase (ADA)	1104	Pf3D7_10_v3: 1,206,302 - 1,207,405 (+)	1.265	8.081	6.391	0.011	0.851	2.4	
PF3D7_1200200	MAL12P1.2, PFL0010c	rifin,PIR protein (RIF)	1137	Pf3D7_12_v3: 26,321 - 27,687 (-)	1.216	7.944	5.214	0.022	0.854	2.3	K13851
PF3D7_0316600	MAL3P6.6	formate-nitrite transporter, putative	930	Pf3D7_03_v3: 669,302 - 670,873 (-)	1.214	11.295	13.358	0.000	0.305	2.3	
PF3D7_0817600	PF08_0058	conserved Plasmodium protein, unknown function	2889	Pf3D7_08_v3: 800,920 - 804,034 (-)	1.163	7.661	3.911	0.048	0.893	2.2	
PF3D7_0404000	MAL4P1.39, PFD0195c	conserved Plasmodium protein, unknown function	300	Pf3D7_04_v3: 223,711 - 224,131 (-)	1.148	7.910	4.480	0.034	0.893	2.2	
PF3D7_1238900	MAL12P1.375, PFL1885c	protein kinase 2 (PK2)	1530	Pf3D7_12_v3: 1,615,323 - 1,616,852 (-)	1.140	8.124	5.307	0.021	0.854	2.2	K13412
PF3D7_1430100	PF14_0280	serine/threonine protein phosphatase 2A activator (PTPA)	960	Pf3D7_14_v3: 1,184,910 - 1,187,042 (+)	1.108	9.222	8.218	0.004	0.703	2.2	
PF3D7_1011500	PF10_0112	conserved Plasmodium membrane protein, unknown function	528	Pf3D7_10_v3: 443,874 - 444,401 (-)	1.090	8.717	6.078	0.014	0.851	2.1	
PF3D7_1216200	MAL12P1.156, PFL0780w	glycerol-3-phosphate dehydrogenase, putative	1104	Pf3D7_12_v3: 648,889 - 650,552 (+)	1.064	8.621	5.374	0.020	0.854	2.1	
PF3D7_0917900	PFI0875w	heat shock protein 70 (HSP70-2)	1959	Pf3D7_09_v3: 737,985 - 740,276	1.046	8.559	6.081	0.014	0.851	2.1	

				(+)						
PF3D7_1217000	MAL12P1.164, PFL0820c	conserved Plasmodium protein, unknown function	429	Pf3D7_12_v3: 674,349 - 674,777 (-)	1.030	9.964	14.135	0.000	0.305	2.0
PF3D7_0406500	MAL4P1.63, PFD0320c	conserved Plasmodium protein, unknown function	9636	Pf3D7_04_v3: 334,282 - 344,054 (-)	0.988	8.301	4.256	0.039	0.893	2.0
PF3D7_0207700	PF02_0073, PFB0345c	serine repeat antigen 4 (SERA4)	2889	Pf3D7_02_v3: 308,847 - 312,155 (-)	0.977	8.649	5.745	0.017	0.851	2.0
PF3D7_1116000	PF11_0168	rhoptry neck protein 4 (RON4)	3606	Pf3D7_11_v3: 602,983 - 606,918 (-)	- 3.317	7.067	11.377	0.001	0.352	0.1
PF3D7_1251600	MAL12P1.493, PFL2480w	conserved Plasmodium protein, unknown function	2139	Pf3D7_12_v3: 2,103,117 - 2,105,255 (+)	- 3.317	7.067	11.377	0.001	0.352	0.1
PF3D7_1205800	MAL12P1.58, PFL0290w	high mobility group protein B3, putative (HMGB3)	6855	Pf3D7_12_v3: 256,367 - 263,731 (+)	- 3.081	6.929	9.065	0.003	0.686	0.1
PF3D7_1334800	MAL13P1.174	MSP7-like protein (MSRP2)	846	Pf3D7_13_v3: 1,413,102 - 1,413,947 (-)	- 2.919	6.848	6.671	0.010	0.851	0.1
PF3D7_1229300	MAL12P1.283, PFL1415w	conserved Plasmodium protein, unknown function	2973	Pf3D7_12_v3: 1,204,991 - 1,207,963 (+)	- 2.799	6.777	6.815	0.009	0.851	0.1
PF3D7_1011000	PF10_0107	inner membrane complex sub-compartment protein 1, putative (ISP1)	435	Pf3D7_10_v3: 429,204 - 430,140 (-)	- 2.742	6.767	4.940	0.026	0.893	0.1
PF3D7_0809500	MAL8P1.114	histone acetyltransferase subunit NuA4, putative	315	Pf3D7_08_v3: 478,512 - 478,826 (-)	- 2.667	7.261	10.361	0.001	0.436	0.2
PF3D7_1249900	MAL12P1.477, PFL2395c	apicoplast dimethyladenosine synthase, putative	1920	Pf3D7_12_v3: 2,041,293 - 2,043,212 (-)	- 2.645	6.708	5.433	0.020	0.854	0.2
PF3D7_0400200	MAL4P1.2	erythrocyte membrane protein 1 (PfEMP1), exon 2, pseudogene	1200	Pf3D7_04_v3: 38,955 - 40,154 (+)	- 2.634	6.697	5.721	0.017	0.851	0.2
PF3D7_0423600	MAL4P1.218	conserved Plasmodium protein, unknown function	4839	Pf3D7_04_v3: 1,061,790 - 1,066,628 (-)	- 2.480	6.623	4.141	0.042	0.893	0.2
PF3D7_0402400	MAL4P1.23, PFD0115c	Plasmodium exported protein, unknown function (GEXP18)	765	Pf3D7_04_v3: 147,490 - 148,380 (-)	- 2.419	6.603	3.938	0.047	0.893	0.2
PF3D7_0509000	MAL5P1.91, PFE0445c	SNAP protein (soluble N-ethylmaleimide-sensitive factor attachment protein), putative	897	Pf3D7_05_v3: 376,855 - 378,452 (-)	- 2.253	7.399	6.315	0.012	0.851	0.2
PF3D7_0217800	PF02_0169, PFB0830w	40S ribosomal protein S26 (RPS26)	324	Pf3D7_02_v3: 734,272 - 734,595 (+)	- 2.231	7.010	6.140	0.013	0.851	0.2

PF3D7_1017200	PF10_0167	enoyl-CoA hydratase-related protein, putative	1593	Pf3D7_10_v3: 692,426 - 694,218 (+)	-2.229	6.996	6.245	0.012	0.851	0.2	
PF3D7_0310700	MAL3P4.24	trafficking protein particle complex subunit 4, putative	438	Pf3D7_03_v3: 463,326 - 464,274 (+)	-2.104	6.943	4.965	0.026	0.893	0.2	
PF3D7_0216000	PF02_0151	DEAD/DEAH box helicase, putative	5994	Pf3D7_02_v3: 658,636 - 664,629 (+)	-2.094	6.928	5.277	0.022	0.854	0.2	
PF3D7_0717700	PF07_0073	serine--tRNA ligase, putative	1620	Pf3D7_07_v3: 764,794 - 766,413 (-)	-1.947	6.861	4.343	0.037	0.893	0.3	K01875
PF3D7_0411900	MAL4P2.51	DNA polymerase alpha	5739	Pf3D7_04_v3: 528,192 - 534,485 (-)	-1.946	6.856	4.343	0.037	0.893	0.3	
PF3D7_1466400	PF14_0633	transcription factor with AP2 domain(s) (ApiAP2)	2442	Pf3D7_14_v3: 2,714,844 - 2,717,285 (-)	-1.946	6.856	4.343	0.037	0.893	0.3	
PF3D7_1339900	PF13_0218	ABC transporter (MDR family), putative (MDR5)	2778	Pf3D7_13_v3: 1,598,401 - 1,601,178 (+)	-1.936	6.851	3.996	0.046	0.893	0.3	
PF3D7_0303900	PFC0176c	phosphatidylethanolamine-binding protein, putative	594	Pf3D7_03_v3: 196,570 - 197,438 (-)	-1.819	7.135	5.171	0.023	0.854	0.3	
PF3D7_0506900	MAL5P1.70, PFE0340c	rhomboid protease ROM4 (ROM4)	2280	Pf3D7_05_v3: 288,776 - 291,055 (-)	-1.619	7.512	6.104	0.013	0.851	0.3	
PF3D7_0505300	MAL5P1.53	UDP-N-acetyl glucosamine:UMP antiporter	1836	Pf3D7_05_v3: 233,815 - 235,650 (+)	-1.545	7.259	4.204	0.040	0.893	0.3	
PF3D7_1411700	PF14_0115	conserved protein, unknown function	2049	Pf3D7_14_v3: 475,249 - 477,297 (+)	-1.544	7.254	4.408	0.036	0.893	0.3	
PF3D7_0931700	PFI1540w	conserved Plasmodium protein, unknown function	2148	Pf3D7_09_v3: 1,270,155 - 1,272,656 (+)	-1.544	7.249	4.408	0.036	0.893	0.3	
PF3D7_1447000	PF14_0448	40S ribosomal protein S5	819	Pf3D7_14_v3: 1,929,931 - 1,931,379 (+)	-1.539	7.479	5.082	0.024	0.870	0.3	K02981
PF3D7_0107900	MAL5P1.91, PFE0445c	conserved Plasmodium protein, unknown function	828	Pf3D7_01_v3: 326,397 - 327,224 (-)	-1.528	7.462	4.976	0.026	0.893	0.3	
PF3D7_1363100	PF13_0333	conserved Plasmodium protein, unknown function	3075	Pf3D7_13_v3: 2,536,315 - 2,539,642 (+)	-1.520	7.457	4.650	0.031	0.893	0.3	
PF3D7_1303500	PF13_0019	sodium/hydrogen exchanger, Na+, H+ antiporter (NHE)	5763	Pf3D7_13_v3: 169,874 - 175,789 (-)	-1.512	7.457	4.269	0.039	0.893	0.4	

PF3D7_0204900	PF02_0046, PFB0220w	ubiE/COQ5 methyltransferase, putative	1065	Pf3D7_02_v3: 211,348 - 212,627 (+)	-1.442	7.412	4.558	0.033	0.893	0.4	
PF3D7_1362400	MAL13P1.310	calpain (Pcalp)	6147	Pf3D7_13_v3: 2,495,924 - 2,502,070 (+)	-1.441	7.413	4.517	0.034	0.893	0.4	
PF3D7_0313100	MAL3P4.5, PFC0550w	ubiquitin-protein ligase, putative (HRD3)	2424	Pf3D7_03_v3: 539,743 - 542,810 (+)	-1.400	7.752	4.493	0.034	0.893	0.4	
PF3D7_1019600	PF10_0189	conserved Plasmodium protein, unknown function	3633	Pf3D7_10_v3: 796,291 - 799,923 (+)	-1.330	7.849	5.584	0.018	0.854	0.4	
PF3D7_1413600	PF14_0134	conserved Plasmodium protein, unknown function	1572	Pf3D7_14_v3: 538,869 - 540,572 (+)	-1.291	7.963	5.751	0.016	0.851	0.4	
PF3D7_1343600	MAL13P1.218	UDP-N-acetylglucosamine pyrophosphorylase, putative	1782	Pf3D7_13_v3: 1,721,904 - 1,723,685 (-)	-1.281	7.515	4.045	0.044	0.893	0.4	K00972
PF3D7_1452800	PF14_0503	conserved Plasmodium protein, unknown function	771	Pf3D7_14_v3: 2,170,461 - 2,171,690 (+)	-1.185	8.340	6.544	0.011	0.851	0.4	
PF3D7_1334700	PF13_0194	MSP7-like protein (MSRP7)	609	Pf3D7_13_v3: 1,411,100 - 1,411,708 (-)	-1.149	8.013	4.747	0.029	0.893	0.5	
PF3D7_1335600	MAL13P1.178	conserved Plasmodium protein, unknown function	1236	Pf3D7_13_v3: 1,454,507 - 1,455,875 (+)	-1.085	8.604	6.784	0.009	0.851	0.5	
PF3D7_1249400	MAL12P1.472, PFL2370c	conserved Plasmodium protein, unknown function	432	Pf3D7_12_v3: 2,016,325 - 2,016,756 (-)	-1.078	7.958	4.047	0.044	0.893	0.5	
PF3D7_0905400	PFI0265c	high molecular weight rhoptry protein 3 (RhopH3)	2694	Pf3D7_09_v3: 270,740 - 274,789 (-)	-0.972	8.748	5.356	0.021	0.854	0.5	
PF3D7_0406700	MAL4P2.03	conserved Plasmodium protein, unknown function	2544	Pf3D7_04_v3: 347,458 - 351,499 (+)	-0.940	8.186	3.889	0.049	0.893	0.5	
PF3D7_0929400	PFI1445w	high molecular weight rhoptry protein 2 (RhopH2)	4137	Pf3D7_09_v3: 1,175,203 - 1,180,762 (+)	-0.893	8.348	3.844	0.050	0.893	0.5	

Supplementary Table 5 - Differentially expressed genes in ring stage treated with cAMP

Gene ID	Gene Symbol	Product Description	Transcript Length	Genomic Location	logFC	logCPM	LR	PValue	FDR	FC	KEEG
PF3D7_0630800	PFF1495w	conserved Plasmodium protein, unknown function	1176	Pf3D7_06_v3: 1,288,237 - 1,290,805 (+)	3	7	9	0,003361847	0,941317067	8	
PF3D7_1024800	PF10_0242	conserved Plasmodium protein, unknown function	4395	Pf3D7_10_v3: 1,034,685 - 1,039,079 (+)	3	7	9	0,003361847	0,941317067	8	
PF3D7_0107500	PFA0375c	lipid/sterol:H+ symporter	4413	Pf3D7_01_v3: 304,362 - 309,257 (-)	3	7	6	0,012199702	0,949347656	7	
PF3D7_1357900	MAL13P1.284	pyrroline-5-carboxylate reductase	789	Pf3D7_13_v3: 2,295,637 - 2,297,156 (+)	3	7	7	0,006489339	0,949347656	7	K00286
PF3D7_0806700	PF08_0114	conserved Plasmodium membrane protein, unknown function	7719	Pf3D7_08_v3: 354,472 - 362,396 (+)	3	7	6	0,012487913	0,949347656	7	
PF3D7_0817300	PF08_0060	asparagine-rich antigen	6708	Pf3D7_08_v3: 782,909 - 789,616 (-)	3	7	5	0,023939682	0,949347656	6	
PF3D7_1442400	PF3D7_1442400	conserved Plasmodium protein, unknown function	6342	Pf3D7_14_v3: 1,717,171 - 1,723,512 (+)	3	7	5	0,023940341	0,949347656	6	
PF3D7_1465900	PF14_0627	40S ribosomal protein S3	666	Pf3D7_14_v3: 2,684,141 - 2,685,534 (+)	2	7	8	0,005130606	0,949347656	5	K02985
PF3D7_0611000	PFF0540c	SNARE associated Golgi protein, putative	864	Pf3D7_06_v3: 463,564 - 464,676 (-)	2	7	4	0,045675279	0,949347656	5	
PF3D7_0932000	PFI1555w	conserved Plasmodium protein, unknown function	309	Pf3D7_09_v3: 1,278,007 - 1,278,896 (+)	2	7	4	0,045675279	0,949347656	5	
PF3D7_1009200	PF10_0089	small subunit rRNA synthesis-associated protein, putative	2748	Pf3D7_10_v3: 374,864 - 377,611 (+)	2	7	4	0,045675321	0,949347656	5	
PF3D7_1220900	PFL1005c	heterochromatin protein 1 (HP1)	801	Pf3D7_12_v3: 831,252 - 832,052 (-)	2	7	4	0,045675928	0,949347656	5	

PF3D7_0212500	PF02_0117, PFB0560w	conserved Plasmodium protein, unknown function	11973	Pf3D7_02_v3: 509,525 - 521,746 (+)	2	7	4	0,045675969	0,949347656	5	
PF3D7_0424600	MAL4P1.229, PFD1170c	Plasmodium exported protein (PHISTb), unknown function	930	Pf3D7_04_v3: 1,113,345 - 1,114,486 (-)	2	7	4	0,045675969	0,949347656	5	
PF3D7_1211800	2277.t00118, MAL12P1.117, PFL0585w	polyubiquitin (PfpUB)	1146	Pf3D7_12_v3: 526,109 - 527,780 (+)	2	7	4	0,045675969	0,949347656	5	K08770
PF3D7_1217400	PFL0840c	conserved Plasmodium protein, unknown function	2565	Pf3D7_12_v3: 686,307 - 688,871 (-)	2	7	4	0,045676011	0,949347656	5	
PF3D7_1201600	2277.t00016, MAL12P1.16, PFL0080c	NIMA related kinase 3 (NEK3)	1044	Pf3D7_12_v3: 101,581 - 102,624 (-)	2	7	6	0,015411685	0,949347656	5	K08857
PF3D7_0913200	PFI0645w	elongation factor 1-beta (EF-1beta)	831	Pf3D7_09_v3: 575,679 - 576,509 (+)	2	7	5	0,033556942	0,949347656	4	K03232
PF3D7_1024500	PF10_0238	conserved Plasmodium protein, unknown function	795	Pf3D7_10_v3: 1,026,120 - 1,026,914 (+)	2	7	4	0,045720653	0,949347656	4	
PF3D7_1255100	2277.t00530, MAL12P1.527, PFL2660w	rifin,PIR protein (RIF)	1053	Pf3D7_12_v3: 2,238,210 - 2,239,417 (+)	2	7	7	0,010470351	0,949347656	4	K13851
PF3D7_0600200	2270.t00002, MAL6P1.316, PFF0010w	erythrocyte membrane protein 1, PfEMP1 (VAR)	8640	Pf3D7_06_v3: 3,503 - 12,835 (+)	2	8	6	0,011023004	0,949347656	4	K13850
PF3D7_0700700	MAL8P1.214	stevor, pseudogene,PIR protein, pseudogene	864	Pf3D7_07_v3: 45,559 - 46,607 (+)	2	9	15	8,38927E-05	0,15228689	4	
PF3D7_0615900	PFF0770c	conserved Plasmodium protein, unknown function	5421	Pf3D7_06_v3: 662,435 - 667,855 (-)	2	8	7	0,00890781	0,949347656	4	
PF3D7_1459900	PF14_0572	conserved Plasmodium membrane protein, unknown function	582	Pf3D7_14_v3: 2,450,207 - 2,450,788 (-)	2	7	4	0,039409026	0,949347656	3	
PF3D7_1115900	PF11_0167	palmitoyltransferase, putative (DHHC9)	882	Pf3D7_11_v3: 600,176 - 602,215 (+)	2	8	6	0,011374316	0,949347656	3	
PF3D7_0922400	PFI1100w	para-aminobenzoic acid synthetase (pBAS)	2940	Pf3D7_09_v3: 908,724 - 912,061 (+)	2	8	4	0,034742957	0,949347656	3	K13950

PF3D7_1040100	PF10_0394	rifin,PIR protein (RIF)	930	Pf3D7_10_v3: 1,602,935 - 1,603,989 (+)	2	8	5	0,025259384	0,949347656	3	K13851
PF3D7_1446400	PF14_0441	pyruvate dehydrogenase E1 component subunit beta (pdhB)	1248	Pf3D7_14_v3: 1,902,916 - 1,904,163 (-)	1	8	5	0,03130712	0,949347656	3	K00162
PF3D7_0317600	PFC0775w	40S ribosomal protein S11, putative (RPS11)	486	Pf3D7_03_v3: 725,015 - 726,130 (+)	1	9	10	0,00157058	0,828782075	3	K02949
PF3D7_0422400	PFD1055w	40S ribosomal protein S19 (RPS19)	513	Pf3D7_04_v3: 1,026,648 - 1,027,399 (+)	1	8	6	0,015422819	0,949347656	3	K02966
PF3D7_0629300	PFF1420w	phosphatidylcholine-sterol acyltransferase, putative (PL)	2592	Pf3D7_06_v3: 1,205,190 - 1,207,781 (+)	1	8	5	0,01963054	0,949347656	2	K00650
PF3D7_1005900	PF10_0060	conserved Plasmodium protein, unknown function	1908	Pf3D7_10_v3: 253,704 - 255,611 (+)	1	8	4	0,044593973	0,949347656	2	
PF3D7_0217800	PFB0830w	40S ribosomal protein S26 (RPS26)	324	Pf3D7_02_v3: 734,272 - 734,595 (+)	1	8	6	0,018666031	0,949347656	2	K02976
PF3D7_1222000	PFL1060c	conserved protein, unknown function	1710	Pf3D7_12_v3: 876,916 - 878,625 (-)	1	9	9	0,002907848	0,941317067	2	K14415
PF3D7_1303500	PF13_0019	sodium/hydrogen exchanger, Na+, H+ antiporter (NHE)	5763	Pf3D7_13_v3: 169,874 - 175,789 (-)	1	8	5	0,029406794	0,949347656	2	
PF3D7_0809600	MAL8P1.113	peptidase family C50, putative	17403	Pf3D7_08_v3: 479,980 - 497,382 (-)	1	8	5	0,020698668	0,949347656	2	
PF3D7_0819600	MAL8P1.62	conserved Plasmodium protein, unknown function	834	Pf3D7_08_v3: 885,198 - 886,136 (-)	-1	8	4	0,046397595	0,949347656	1	
PF3D7_1023900	PF10_0232	chromodomain-helicase-DNA-binding protein 1 homolog, putative (CHD1)	9987	Pf3D7_10_v3: 997,475 - 1,007,461 (-)	-1	8	4	0,036456766	0,949347656	0	K11367
PF3D7_0913300	PFI0650c	conserved Plasmodium protein, unknown function	1941	Pf3D7_09_v3: 576,960 - 579,026 (-)	-1	9	5	0,027032137	0,949347656	0	K00858
PF3D7_0114000	MAL1P4.13, PFA0675w	exported protein family 1 (EPF1)	4392	Pf3D7_01_v3: 542,480 - 546,983 (+)	-1	8	5	0,030435594	0,949347656	0	

PF3D7_1238900	MAL12P1.375, PFL1885c	protein kinase 2 (PK2)	1530	Pf3D7_12_v3: 1,615,323 - 1,616,852 (-)	-1	8	4	0,048987282	0,949347656	0	
PF3D7_1308200	PF13_0044	carbamoyl phosphate synthetase (cpsSII)	7128	Pf3D7_13_v3: 361,166 - 368,495 (-)	-1	8	4	0,046215329	0,949347656	0	K01954
PF3D7_1452600	PF14_0501	conserved Plasmodium protein, unknown function	10044	Pf3D7_14_v3: 2,155,736 - 2,166,467 (+)	-1	8	5	0,021358347	0,949347656	0	
PF3D7_0115500	PFA0755w	erythrocyte membrane protein 1 (PfEMP1), pseudogene	1299	Pf3D7_01_v3: 600,692 - 601,992 (+)	-1	8	4	0,039015556	0,949347656	0	
PF3D7_1371800	MAL13P1.110	Plasmodium exported protein, unknown function	1323	Pf3D7_13_v3: 2,824,302 - 2,825,852 (-)	-1	8	6	0,012845233	0,949347656	0	
PF3D7_1141800	PF11_0429	phd finger protein, putative	1185	Pf3D7_11_v3: 1,672,519 - 1,674,557 (-)	-2	7	5	0,02603107	0,949347656	0	
PF3D7_1331400	PF13_0168	CPW-WPC family protein	1446	Pf3D7_13_v3: 1,310,771 - 1,312,216 (+)	-2	7	5	0,026023987	0,949347656	0	
PF3D7_1428700	PF14_0266	conserved protein, unknown function	1302	Pf3D7_14_v3: 1,129,470 - 1,130,771 (-)	-2	8	5	0,022398187	0,949347656	0	
PF3D7_1249400	PFL2370c	conserved Plasmodium protein, unknown function	432	Pf3D7_12_v3: 2,016,325 - 2,016,756 (-)	-2	8	6	0,012295873	0,949347656	0	
PF3D7_0822000	PF08_0038	mitochondrial ribosomal protein L4 precursor, putative	1707	Pf3D7_08_v3: 978,798 - 980,634 (+)	-2	7	6	0,017662506	0,949347656	0	
PF3D7_1343000	MAL13P1.214	phosphoethanolamine N-methyltransferase (PMT)	801	Pf3D7_13_v3: 1,696,894 - 1,698,113 (+)	-2	7	4	0,04420495	0,949347656	0	K05929
PF3D7_0403800	MAL4P1.37, PFD0185c	alpha/beta hydrolase, putative	2205	Pf3D7_04_v3: 211,650 - 213,854 (-)	-2	7	5	0,028921984	0,949347656	0	
PF3D7_0932100	PFI1560c	protein MAM3, putative	3825	Pf3D7_09_v3: 1,279,219 - 1,283,879 (-)	-2	7	5	0,028921533	0,949347656	0	K16302
PF3D7_1130000	PF11_0311	phosphoacetylglucosamine mutase, putative (PAGM)	2823	Pf3D7_11_v3: 1,159,366 - 1,162,188 (-)	-2	7	5	0,028921533	0,949347656	0	K01836

PF3D7_0900100	PFI0005w	erythrocyte membrane protein 1, PfEMP1 (VAR)	6813	Pf3D7_09_v3: 20,080 - 27,885 (+)	-2	7	4	0,036496476	0,949347656	0	K13850
PF3D7_0828300	PF08_0010	conserved Plasmodium protein, unknown function	1659	Pf3D7_08_v3: 1,221,025 - 1,222,683 (-)	-2	7	6	0,016314663	0,949347656	0	
PF3D7_0828500	PF08_0009	translation initiation factor EIF-2b alpha subunit, putative	1029	Pf3D7_08_v3: 1,226,750 - 1,228,409 (-)	-2	7	6	0,01631433	0,949347656	0	K03239
PF3D7_1229000	PFL1405w	conserved Plasmodium membrane protein, unknown function	5505	Pf3D7_12_v3: 1,186,415 - 1,191,919 (+)	-2	7	4	0,047405891	0,949347656	0	
PF3D7_0424700	PFD1175w	serine/threonine protein kinase, FIKK family (FIKK4.2)	3669	Pf3D7_04_v3: 1,118,430 - 1,122,414 (+)	-2	7	4	0,047405818	0,949347656	0	
PF3D7_1126500	PF11_0275	conserved Plasmodium protein, unknown function	4107	Pf3D7_11_v3: 1,033,062 - 1,037,168 (+)	-2	7	4	0,047405488	0,949347656	0	
PF3D7_0415300	MAL4P1.143, PFD0740w	cdc2-related protein kinase 3 (CRK3)	4020	Pf3D7_04_v3: 681,810 - 685,968 (+)	-2	7	4	0,047405414	0,949347656	0	
PF3D7_1235700	PFL1725w	ATP synthase subunit beta, mitochondrial	1608	Pf3D7_12_v3: 1,487,084 - 1,488,691 (+)	-2	7	4	0,047405064	0,949347656	0	K02133
PF3D7_1008200	PF10_0080	endonuclease, putative	1167	Pf3D7_10_v3: 343,733 - 344,899 (-)	-2	7	4	0,04740499	0,949347656	0	
PF3D7_1302800	PF13_0014	40S ribosomal protein S7, putative	585	Pf3D7_13_v3: 150,783 - 151,635 (-)	-2	7	6	0,013415942	0,949347656	0	K02993
PF3D7_1457600	PF14_0549	conserved Plasmodium protein, unknown function	1662	Pf3D7_14_v3: 2,363,578 - 2,365,370 (+)	-3	7	4	0,038193212	0,949347656	0	
PF3D7_1213300	PFL0645c	conserved Plasmodium protein, unknown function	336	Pf3D7_12_v3: 578,470 - 578,917 (-)	-3	7	5	0,025000608	0,949347656	0	
PF3D7_0103400	MAL1P1.74b, PFA0170c	zinc-carboxypeptidase, putative	4863	Pf3D7_01_v3: 147,915 - 152,777 (-)	-3	7	5	0,025000533	0,949347656	0	
PF3D7_0826100	MAL8P1.23	E3 ubiquitin-protein ligase, putative	25776	Pf3D7_08_v3: 1,113,369 - 1,139,144 (-)	-3	7	5	0,025000533	0,949347656	0	K10592

PF3D7_0632500	PFF1580c	erythrocyte membrane protein 1, PfEMP1 (VAR)	11865	Pf3D7_06_v3: 1,353,946 - 1,366,430 (-)	-3	7	5	0,02500016	0,949347656	0	K13850
PF3D7_0819000	MAL8P1.64	conserved Plasmodium protein, unknown function	3942	Pf3D7_08_v3: 863,505 - 868,137 (-)	-3	7	5	0,024999697	0,949347656	0	
PF3D7_0420000	PFD0970c	zinc finger protein, putative	10113	Pf3D7_04_v3: 894,612 - 904,724 (-)	-3	7	6	0,01312126	0,949347656	0	
PF3D7_0316800	PFC0735w	40S ribosomal protein S15A, putative	393	Pf3D7_03_v3: 677,842 - 678,474 (+)	-3	7	5	0,026693593	0,949347656	0	K02957
PF3D7_0617600	PF3D7_0617600	stevor,PIR protein	909	Pf3D7_06_v3: 738,259 - 739,265 (-)	-3	7	7	0,006861103	0,949347656	0	
PF3D7_1200300	PFL0015c	rifin,PIR protein (RIF)	1038	Pf3D7_12_v3: 30,078 - 31,261 (-)	-3	7	7	0,008685255	0,949347656	0	K13851
PF3D7_0315400	PFC0670c		771	Pf3D7_03_v3: 630,611 - 631,381 (-)	-3,288585993	7	10	0,001292395	0,828782075	0,102338011	

Supplementary Table 6 -Differentially expressed genes in schizont stage treated with cAMP

Gene ID	Gene Symbol	Product Description	Transcript Length	Genomic Location	logFC	logCPM	LR	PValue	FDR	FC	KEGG
PF3D7_0930600	PFI1490c	peptidyl-prolyl cis-trans isomerase (CYP72)	1830	Pf3D7_09_v3 : 1,218,393 - 1,221,000 (-)	32.562	62.163	77.807	0.0053	0.4008	9.6	K10598
PF3D7_0607700	2270.t00236, MAL6P1.80, PFF0380w	conserved Plasmodium protein, unknown function	8259	Pf3D7_06_v3 : 320,740 - 328,998 (+)	29.978	61.206	82.788	0.0040	0.3671	8.0	
PF3D7_1316600	MAL13P1.86, PF13_0092	choline-phosphate cytidylyltransferase (CCT)	2691	Pf3D7_13_v3 : 691,014 - 694,125 (+)	29.615	61.114	73.314	0.0068	0.4167	7.8	K00968
PF3D7_0405500	MAL4P1.53, PFD0265c, PFD0270c	conserved Plasmodium protein, unknown function	675	Pf3D7_04_v3 : 301,259 - 302,267 (-)	28.498	60.457	71.242	0.0076	0.4445	7.2	
PF3D7_1473600	PF14_0705	conserved Plasmodium protein, unknown function	345	Pf3D7_14_v3 : 2,997,600 - 2,997,944 (-)	28.472	60.246	69.667	0.0083	0.4544	7.2	
PF3D7_1142100	PF11_0433	conserved Plasmodium protein, unknown function	8217	Pf3D7_11_v3 : 1,681,868 - 1,690,084 (-)	28.472	60.246	69.628	0.0083	0.4544	7.2	
PF3D7_1014100	PF10_0138	conserved Plasmodium protein, unknown function	4368	Pf3D7_10_v3 : 555,160 - 559,527 (+)	28.054	60.154	57.395	0.0166	0.5551	7.0	
PF3D7_0801500	PF08_0135	conserved Plasmodium protein, unknown function	1650	Pf3D7_08_v3 : 106,593 - 108,242 (-)	27.465	59.732	55.241	0.0188	0.5700	6.7	K14788
PF3D7_1133200	MAL5P1.225, PFE1125w	50S ribosomal protein L17, apicoplast, putative (RPL17)	501	Pf3D7_05_v3 : 937,231 - 938,037 (+)	26.849	59.451	59.917	0.0144	0.5389	6.4	
PF3D7_0522500	PF11_0342	conserved Plasmodium protein, unknown function	6219	Pf3D7_11_v3 : 1,281,284 - 1,287,633 (-)	26.849	59.451	59.917	0.0144	0.5389	6.4	
PF3D7_0920200	3D7surf1.2, MAL1P12a, MAL1P3.12, MAL1P3.12a, PFA0650w, PFA0655, PFA0655w	surface-associated interspersed protein 1.2 (SURFIN 1.2), pseudogene (SURF1.2)	5682	Pf3D7_01_v3 : 512,086 - 517,855 (+)	24.986	58.606	48.862	0.0271	0.5847	5.7	

PF3D7_0113600	PFI0990c	CS domain protein, putative	2952	Pf3D7_09_v3 : 827,730 - 830,681 (-)	24.986	58.606	48.862	0.0271	0.5847	5.7	
PF3D7_1131500	MAL5P1.113, PFE0560c	MORN repeat protein, putative	4254	Pf3D7_05_v3 : 474,487 - 478,882 (-)	24.985	58.727	48.860	0.0271	0.5847	5.7	
PF3D7_0511300	PF11_0325	conserved Plasmodium protein, unknown function	771	Pf3D7_11_v3 : 1,210,686 - 1,211,456 (-)	24.985	58.727	48.860	0.0271	0.5847	5.7	
PF3D7_1236700	2277.t00355, MAL12P1.353, PFL1775c	S-adenosyl-methyltransferase, putative	1527	Pf3D7_12_v3 : 1,529,821 - 1,531,347 (-)	24.985	58.727	48.860	0.0271	0.5847	5.7	
PF3D7_0624100	2270.t00127, MAL6P1.188, PFF1160w	conserved Plasmodium protein, unknown function	819	Pf3D7_06_v3 : 983,946 - 984,989 (+)	24.893	58.512	46.445	0.0312	0.6087	5.6	
PF3D7_0908600	PFI0415c	ribosomal RNA methyltransferase, putative	1524	Pf3D7_09_v3 : 399,202 - 400,725 (-)	24.398	66.986	97.861	0.0018	0.2428	5.4	K14864
PF3D7_1458500	PF14_0558	spindle assembly abnormal protein 4, putative (SAS4)	4410	Pf3D7_14_v3 : 2,400,515 - 2,405,411 (+)	24.052	63.198	76.255	0.0058	0.4167	5.3	
PF3D7_1439900	PF14_0378	triosephosphate isomerase (TIM)	747	Pf3D7_14_v3 : 1,622,576 - 1,623,612 (+)	23.548	63.135	61.813	0.0129	0.5389	5.1	K01803
PF3D7_0407100	MAL4P2.07, PFD0350w	methyltransferase, putative	1002	Pf3D7_04_v3 : 362,301 - 363,442 (+)	22.802	66.007	89.722	0.0027	0.2752	4.9	
PF3D7_1354400	MAL13P1.271	V-type proton ATPase 21 kDa proteolipid subunit, putative	546	Pf3D7_13_v3 : 2,169,314 - 2,170,416 (-)	21.911	65.971	79.108	0.0049	0.3823	4.6	
PF3D7_0409200	MAL4P2.28, PFD0455w	40S ribosomal processing protein, putative	1449	Pf3D7_04_v3 : 438,887 - 440,335 (+)	21.669	65.514	73.319	0.0068	0.4167	4.5	K11806
PF3D7_0614800	2270.t00038, MAL6P1.278, PFF0715c	endonuclease III homologue, putative	1314	Pf3D7_06_v3 : 615,297 - 616,610 (-)	21.540	68.409	95.235	0.0020	0.2428	4.5	K10773
PF3D7_1039000	PF10_0380	serine/threonine protein kinase, FIKK family (FIKK10.2)	2742	Pf3D7_10_v3 : 1,568,709 - 1,571,815 (+)	20.920	64.951	73.915	0.0066	0.4167	4.3	
PF3D7_0409600	MAL4P2.32, PFD0470c, PFD0475c	replication protein A1, large subunit (RPA1)	3438	Pf3D7_04_v3 : 452,134 - 455,571 (-)	20.920	65.149	73.910	0.0066	0.4167	4.3	

PF3D7_1366400	PF13_0348	rhoptry protein (Rhop148)	3789	Pf3D7_13_v3 : 2,655,277 - 2,659,065 (-)	20.461	61.264	42.950	0.0382	0.6130	4.1	
PF3D7_0514600	MAL5P1.146, PFE0730c	ribose 5-phosphate epimerase, putative	711	Pf3D7_05_v3 : 607,105 - 608,451 (-)	20.243	64.693	40.542	0.0441	0.6306	4.1	K01807
PF3D7_0517000	PFE0850c, PFE0850w	60S ribosomal protein L12, putative	498	Pf3D7_05_v3 : 715,765 - 716,675 (-)	20.040	61.172	45.611	0.0327	0.6092	4.0	K02870
PF3D7_0113200	MAL1P3.08, PFA0630c	Plasmodium exported protein, unknown function	1779	Pf3D7_01_v3 : 500,959 - 502,868 (-)	20.000	61.291	46.116	0.0318	0.6087	4.0	
PF3D7_0317800	MAL3P6.16, PFC0785c	proteasome regulatory protein, putative	678	Pf3D7_03_v3 : 738,205 - 738,882 (-)	19.975	60.990	43.453	0.0371	0.6130	4.0	K06693
PF3D7_1240600	2277.t00392, MAL12P1.390, PFL1960w	erythrocyte membrane protein 1, PfEMP1 (VAR)	7080	Pf3D7_12_v3 : 1,719,574 - 1,727,456 (+)	19.891	61.107	43.683	0.0366	0.6116	4.0	K13850
PF3D7_1106200	PF11_0073	conserved Plasmodium protein, unknown function	2046	Pf3D7_11_v3 : 260,867 - 263,331 (+)	18.860	64.095	52.296	0.0222	0.5711	3.7	
PF3D7_0803500	MAL8P1.144	AAA family ATPase, putative	4404	Pf3D7_08_v3 : 217,888 - 222,291 (+)	18.740	64.006	55.138	0.0189	0.5700	3.7	
PF3D7_1414300	PF14_0141	60S ribosomal protein L10, putative	660	Pf3D7_14_v3 : 570,802 - 571,461 (+)	17.994	74.888	126.597	0.0004	0.1057	3.5	K02866
PF3D7_0926300	PFI1285w, PFI1290w	protein kinase, putative	3540	Pf3D7_09_v3 : 1,064,752 - 1,070,317 (+)	17.511	63.102	46.223	0.0316	0.6087	3.4	
PF3D7_0917600	PFI0860c	pre-mRNA-splicing factor ATP-dependent RNA helicase PRP43, putative (PRP43)	2463	Pf3D7_09_v3 : 729,674 - 732,136 (-)	16.482	70.372	56.150	0.0178	0.5589	3.1	K12820
PF3D7_0905600	PFI0275w	conserved Plasmodium protein, unknown function	3663	Pf3D7_09_v3 : 280,207 - 284,102 (+)	16.193	73.851	104.736	0.0012	0.2217	3.1	
PF3D7_1033600	PF10_0327	myb2 transcription factor, putative (Myb2)	2748	Pf3D7_10_v3 : 1,344,150 - 1,346,897 (-)	15.995	65.256	47.471	0.0293	0.6087	3.0	K12860
PF3D7_1356500	MAL13P1.276	conserved Plasmodium protein, unknown function	588	Pf3D7_13_v3 : 2,239,261 - 2,240,004 (+)	15.995	65.256	47.471	0.0293	0.6087	3.0	

PF3D7_1458300	PF14_0556	conserved Plasmodium protein, unknown function	4587	Pf3D7_14_v3 : 2,391,783 - 2,396,831 (-)	15.909	65.082	45.383	0.0331	0.6092	3.0	
PF3D7_0416900	MAL4P1.158, PFD0815c, PFD0815c:exon:2	conserved Plasmodium protein, unknown function	7269	Pf3D7_04_v3 : 732,452 - 739,872 (-)	15.590	70.949	55.351	0.0186	0.5700	2.9	
PF3D7_1437800	PF14_0358	41-2 protein antigen precursor,trafficking protein particle complex subunit 5, putative (TRAPPC5)	555	Pf3D7_14_v3 : 1,530,276 - 1,530,830 (-)	15.357	68.480	41.966	0.0405	0.6149	2.9	
PF3D7_0401800	MAL4P1.16, PFD0080c	Plasmodium exported protein (PHISTb), unknown function (PfD80)	1683	Pf3D7_04_v3 : 103,742 - 105,603 (-)	15.178	77.748	118.13 4	0.0006	0.1408	2.9	
PF3D7_1025500	PF10_0249, PF10_0250, PF10_0251	conserved Plasmodium protein, unknown function	17304	Pf3D7_10_v3 : 1,067,652 - 1,086,992 (-)	15.124	68.556	57.786	0.0162	0.5551	2.9	
PF3D7_1315500	MAL13P1.81	conserved Plasmodium protein, unknown function	552	Pf3D7_13_v3 : 652,794 - 653,838 (-)	15.046	68.687	59.176	0.0150	0.5424	2.8	
PF3D7_1420000	PF14_0194	splicing factor 3B subunit 4, putative (SF3B4)	1455	Pf3D7_14_v3 : 828,904 - 830,773 (-)	15.033	66.661	48.518	0.0276	0.5847	2.8	K12831
PF3D7_0706100	MAL7P1.203	conserved Plasmodium protein, unknown function	4590	Pf3D7_07_v3 : 297,506 - 303,059 (-)	14.850	66.821	46.347	0.0313	0.6087	2.8	
PF3D7_0507900	MAL5P1.80, PFE0390w	conserved Plasmodium protein, unknown function	354	Pf3D7_05_v3 : 327,656 - 328,316 (+)	14.152	66.164	40.265	0.0448	0.6306	2.7	
PF3D7_1323000	PF13_0128	beta-hydroxyacyl-ACP dehydratase (FabZ)	693	Pf3D7_13_v3 : 963,661 - 964,690 (-)	13.897	71.254	63.739	0.0116	0.5149	2.6	K02372
PF3D7_0935800	PFI1730w	cytoadherence linked asexual protein 9 (CLAG9)	4023	Pf3D7_09_v3 : 1,413,840 - 1,419,754 (+)	13.861	66.129	38.445	0.0499	0.6447	2.6	
PF3D7_0318500	MAL3P6.23, PFC0820w	conserved Plasmodium protein, unknown function	14946	Pf3D7_03_v3 : 767,815 - 782,760 (+)	13.804	68.010	39.067	0.0481	0.6447	2.6	
PF3D7_1113100	PF11_0139	protein tyrosine phosphatase (PRL)	657	Pf3D7_11_v3 : 511,105 - 511,761 (-)	13.505	72.840	52.298	0.0222	0.5711	2.6	K18041
PF3D7_1412200	PF14_0121	conserved Plasmodium protein, unknown function	2283	Pf3D7_14_v3 : 487,776 -	12.903	69.284	46.807	0.0305	0.6087	2.4	

				490,380 (-)							
PF3D7_0525800	MAL5P1.257, PFE1285w	inner membrane complex protein 1g, putative (IMC1g)	903	Pf3D7_05_v3 : 1,071,247 - 1,072,149 (+)	12.549	70.323	49.430	0.0262	0.5847	2.4	
PF3D7_0718400	MAL7P1.93	mitochondrial ribosomal protein S8 precursor, putative	390	Pf3D7_07_v3 : 822,438 - 823,015 (+)	12.508	75.416	52.465	0.0220	0.5711	2.4	
PF3D7_0602500	2270.t00290, MAL6P1.28, PFF0120w	geranylgeranyltransferase, putative	1140	Pf3D7_06_v3 : 105,503 - 106,642 (+)	10.932	75.198	43.619	0.0368	0.6116	2.1	K05956
PF3D7_0911100	PFI0540w	conserved Plasmodium protein, unknown function	3498	Pf3D7_09_v3 : 504,412 - 508,130 (+)	10.822	72.696	41.453	0.0418	0.6277	2.1	
PF3D7_1366500	PF13_0349	nucleoside diphosphate kinase (NDK)	450	Pf3D7_13_v3 : 2,661,573 - 2,662,022 (+)	10.797	72.943	42.903	0.0383	0.6130	2.1	K00940
PF3D7_0301100	MAL3P8.19, PFC0055w	Plasmodium exported protein (hyp13), unknown function	771	Pf3D7_03_v3 : 76,494 - 77,474 (+)	10.765	72.107	40.719	0.0436	0.6306	2.1	
PF3D7_0211800	PF02_0110, PFB0525w	asparagine--tRNA ligase (AsnRS)	1833	Pf3D7_02_v3 : 475,242 - 477,074 (+)	10.633	72.645	38.838	0.0488	0.6447	2.1	K01893
PF3D7_1021800	PF10_0212, PF10_0212a	schizont egress antigen-1 (SEA1)	6225	Pf3D7_10_v3 : 896,865 - 903,608 (-)	10.508	73.808	45.062	0.0338	0.6092	2.1	
PF3D7_0703300	MAL7P1.11	conserved Plasmodium protein, unknown function	441	Pf3D7_07_v3 : 128,540 - 128,980 (+)	10.237	79.353	68.529	0.0088	0.4682	2.0	
PF3D7_1470200	PF14_0669	conserved Plasmodium protein, unknown function	546	Pf3D7_14_v3 : 2,877,586 - 2,878,748 (-)	0.9931	77.095	42.594	0.0390	0.6130	2.0	
PF3D7_0406200	MAL4P1.61, PFD0310w	sexual stage-specific protein precursor (Pfs16)	474	Pf3D7_04_v3 : 329,216 - 329,689 (+)	0.9795	87.308	92.216	0.0024	0.2481	2.0	
PF3D7_0918900	PFI0925w	gamma-glutamylcysteine synthetase (gammaGCS)	3192	Pf3D7_09_v3 : 777,558 - 780,749 (+)	-0.8651	93.203	97.210	0.0018	0.2428	0.5	K11204
PF3D7_1018900	PF10_0182	conserved Plasmodium protein, unknown function	2151	Pf3D7_10_v3 : 752,760 - 754,910 (-)	-0.8959	84.480	68.175	0.0090	0.4682	0.5	
PF3D7_1478900	PF14TR008, PF14TR008:ncRNA	unspecified product	3988	Pf3D7_14_v3 : 3,247,032 -	-0.9111	82.379	63.280	0.0119	0.5209	0.5	

	, PF14TR009, PF14TR010, PF14TR011			3,251,019 (+)							
PF3D7_1035100	PF10_0342	probable protein, unknown function	1686	Pf3D7_10_v3 : 1,391,445 - 1,393,130 (+)	-0.9274	77.280	42.499	0.0393	0.6130	0.5	
PF3D7_1215200	2277.t00148, MAL12P1.147, PFL0735w	peptidyl-prolyl cis-trans isomerase (CYP32)	843	Pf3D7_12_v3 : 627,361 - 628,203 (+)	-0.9486	88.530	96.253	0.0019	0.2428	0.5	K01802
PF3D7_0420300	MAL4P1.192, PFD0985w	transcription factor with AP2 domain(s) (ApiAP2)	10422	Pf3D7_04_v3 : 917,990 - 928,411 (+)	-0.9512	78.456	54.833	0.0192	0.5711	0.5	
PF3D7_0709100	PF07_0035	Cg1 protein	3747	Pf3D7_07_v3 : 408,215 - 411,961 (+)	-0.9692	77.075	46.095	0.0318	0.6087	0.5	
PF3D7_0627800	2270.t00165, MAL6P1.150, PFF1350c	acetyl-CoA synthetase, putative (ACS)	2994	Pf3D7_06_v3 : 1,114,544 - 1,117,537 (-)	-0.9727	89.874	105.29 9	0.0012	0.2217	0.5	K01895
PF3D7_0218400	PF02_0174, PFB0860c	DEAD/DEAH box ATP-dependent RNA helicase, putative	1689	Pf3D7_02_v3 : 750,049 - 751,737 (-)	-0.9800	76.387	48.844	0.0271	0.5847	0.5	K14777
PF3D7_1030200	PF10_0295	conserved Plasmodium protein, unknown function	1353	Pf3D7_10_v3 : 1,230,561 - 1,231,913 (-)	-0.9863	77.887	56.001	0.0180	0.5589	0.5	
PF3D7_1450600	PF14_0481	conserved Plasmodium protein, unknown function	1764	Pf3D7_14_v3 : 2,075,586 - 2,077,349 (-)	-0.9892	79.037	59.580	0.0147	0.5405	0.5	
PF3D7_0612800	2270.t00020, MAL6P1.298, PFF0620c	6-cysteine protein (P12p)	1116	Pf3D7_06_v3 : 528,060 - 529,175 (-)	-10.293	79.102	57.607	0.0164	0.5551	0.5	
PF3D7_1217600	2277.t00170, MAL12P1.170, PFL0850w	anaphase promoting complex subunit 10, putative	897	Pf3D7_12_v3 : 695,559 - 696,455 (+)	-10.636	85.185	104.97 2	0.0012	0.2217	0.5	K03357
PF3D7_1222100	2277.t00213, MAL12P1.213, PFL1065c	conserved Plasmodium protein, unknown function	1557	Pf3D7_12_v3 : 879,630 - 881,186 (-)	-10.810	76.418	57.020	0.0169	0.5551	0.5	
PF3D7_0624700	2270.t00133, MAL6P1.182, PFF1190c	N-acetylglucosaminylphosphatidylinositol deacetylase, putative	744	Pf3D7_06_v3 : 1,009,457 - 1,010,718 (-)	-10.817	74.368	53.611	0.0206	0.5711	0.5	K03434
PF3D7_0727800	PF07_0115	cation transporting ATPase, putative	5757	Pf3D7_07_v3 : 1,175,944 - 1,181,700 (+)	-10.953	81.806	95.238	0.0020	0.2428	0.5	K14950

PF3D7_1468800	PF14_0656	splicing factor U2AF large subunit, putative (U2AF2)	2502	Pf3D7_14_v3 : 2,827,942 - 2,830,443 (-)	-10.992	70.301	39.445	0.0470	0.6447	0.5	K12837
PF3D7_1344800	MAL13P1.221, PF13_0240	aspartate carbamoyltransferase (ATCase)	1128	Pf3D7_13_v3 : 1,796,274 - 1,797,709 (+)	-11.358	104.666	420.90 5	0.0000	0.0000	0.5	K00609
PF3D7_1010300	PF10_0100	succinate dehydrogenase subunit 4, putative (SDH4)	357	Pf3D7_10_v3 : 414,732 - 415,328 (+)	-11.586	73.660	40.266	0.0448	0.6306	0.4	
PF3D7_1446900	PF14_0447	glutaminyl-peptide cyclotransferase, putative	1152	Pf3D7_14_v3 : 1,924,425 - 1,926,650 (-)	-11.599	71.782	49.607	0.0259	0.5847	0.4	
PF3D7_1428700	PF14_0266	conserved protein, unknown function	1302	Pf3D7_14_v3 : 1,129,470 - 1,130,771 (-)	-12.638	70.080	49.324	0.0264	0.5847	0.4	
PF3D7_1460500	PF14_0577	conserved Plasmodium protein, unknown function	4899	Pf3D7_14_v3 : 2,466,091 - 2,470,989 (+)	-12.641	70.104	49.349	0.0263	0.5847	0.4	
PF3D7_0912700	PFI0620w	conserved Plasmodium protein, unknown function	258	Pf3D7_09_v3 : 562,677 - 562,934 (+)	-12.917	68.588	45.839	0.0323	0.6087	0.4	
PF3D7_0831600	MAL7P1.229	cytoadherence linked asexual protein 8 (CLAG8)	4185	Pf3D7_08_v3 : 1,358,314 - 1,363,618 (+)	-13.188	73.919	73.792	0.0066	0.4167	0.4	
PF3D7_1476600	PF14_0736	Plasmodium exported protein, unknown function	2868	Pf3D7_14_v3 : 3,151,970 - 3,154,923 (+)	-13.190	72.784	44.468	0.0350	0.6116	0.4	
PF3D7_0723300	MAL7P1.125	conserved Plasmodium protein, unknown function	2643	Pf3D7_07_v3 : 975,766 - 978,408 (+)	-13.279	67.069	42.437	0.0394	0.6130	0.4	
PF3D7_0800400	null	rifin,PIR protein (RIF)	993	Pf3D7_08_v3 : 52,760 - 53,982 (-)	-13.281	67.178	42.450	0.0394	0.6130	0.4	
PF3D7_1311500	PF13_0063	26S protease regulatory subunit 7, putative (RPT1)	1263	Pf3D7_13_v3 : 489,927 - 491,609 (-)	-13.372	70.478	56.612	0.0173	0.5564	0.4	K03061
PF3D7_1022200	PF10_0215	conserved Plasmodium membrane protein, unknown function	1890	Pf3D7_10_v3 : 926,831 - 929,020 (-)	-13.770	65.723	39.174	0.0478	0.6447	0.4	
PF3D7_0802300	PF08_0130	rRNA processing WD-repeat protein, putative	3366	Pf3D7_08_v3 : 167,069 - 170,434 (-)	-14.064	70.817	64.134	0.0113	0.5149	0.4	K14558

PF3D7_1228800	2277.t00279, MAL12P1.279, PFL1395c	conserved Plasmodium protein, unknown function	9630	Pf3D7_12_v3 : 1,169,557 - 1,179,186 (-)	-14.147	70.301	44.210	0.0355	0.6116	0.4	
PF3D7_1015500	PF10_0152	conserved Plasmodium protein, unknown function	1848	Pf3D7_10_v3 : 624,048 - 625,895 (-)	-14.197	67.825	50.045	0.0253	0.5847	0.4	
PF3D7_0215700	PF02_0148, PFB0715w	DNA-directed RNA polymerase II second largest subunit, putative	4023	Pf3D7_02_v3 : 645,929 - 650,085 (+)	-14.553	73.871	79.919	0.0047	0.3757	0.4	K03010
PF3D7_0202900	PF02_0028, PFB0140w	palmitoyltransferase, putative (DHHC12)	864	Pf3D7_02_v3 : 138,927 - 141,192 (+)	-14.610	73.625	65.981	0.0102	0.4964	0.4	
PF3D7_0616800	2270.t00056, MAL6P1.258, PFF0815w	malate:quinone oxidoreductase, putative	1566	Pf3D7_06_v3 : 695,929 - 697,494 (+)	-14.727	68.050	48.540	0.0276	0.5847	0.4	K00116
PF3D7_0603200	2270.t00283, MAL6P1.35, PFF0155w	mitochondrial chaperone BCS1, putative	1416	Pf3D7_06_v3 : 128,621 - 130,036 (+)	-14.815	66.013	46.982	0.0302	0.6087	0.4	K08900
PF3D7_0803000	PF08_0128	peptidyl-prolyl cis-trans isomerase (CYP81)	2034	Pf3D7_08_v3 : 196,581 - 198,614 (+)	-14.886	66.516	43.699	0.0366	0.6116	0.4	
PF3D7_0305200	MAL3P2.16, PFC0235w	conserved Plasmodium protein, unknown function	3420	Pf3D7_03_v3 : 251,242 - 254,661 (+)	-15.680	64.407	44.179	0.0356	0.6116	0.3	
PF3D7_1220600	2277.t00198, MAL12P1.198, PFL0990w	conserved Plasmodium protein, unknown function	1017	Pf3D7_12_v3 : 820,586 - 822,198 (+)	-15.740	80.088	134.307	0.0002	0.0856	0.3	
PF3D7_1023300	PF10_0226	conserved Plasmodium protein, unknown function	240	Pf3D7_10_v3 : 984,228 - 985,330 (-)	-16.713	67.196	63.762	0.0116	0.5149	0.3	
PF3D7_1434300	PF14_0324	Hsp70/Hsp90 organizing protein (HOP)	1695	Pf3D7_14_v3 : 1,372,903 - 1,374,597 (+)	-16.897	62.553	40.353	0.0446	0.6306	0.3	K09553
PF3D7_1135300	PF11_0363	conserved Plasmodium membrane protein, unknown function	1230	Pf3D7_11_v3 : 1,381,015 - 1,382,244 (-)	-17.649	68.036	66.697	0.0098	0.4922	0.3	
PF3D7_1217400	2277.t00168, MAL12P1.168, PFL0840c	conserved Plasmodium protein, unknown function	2565	Pf3D7_12_v3 : 686,307 - 688,871 (-)	-18.791	63.356	47.268	0.0297	0.6087	0.3	
PF3D7_0307200	MAL3P2.29, PFC0300c	60S ribosomal protein L7, putative	774	Pf3D7_03_v3 : 314,976 - 316,236 (-)	-19.141	60.367	40.323	0.0446	0.6306	0.3	K02937

PF3D7_1476800	MAL13P1.480	histidine-rich protein III (HRPIII)	828	Pf3D7_13_v3 : 2,840,727 - 2,841,703 (-)	-19.141	60.367	40.323	0.0446	0.6306	0.3	
PF3D7_1372200	PF14_0738	lysophospholipase, putative	1116	Pf3D7_14_v3 : 3,159,487 - 3,160,602 (+)	-19.141	60.367	40.323	0.0446	0.6306	0.3	
PF3D7_0821300	PF08_0042	ATP-dependent RNA helicase prh1, putative	2604	Pf3D7_08_v3 : 958,531 - 961,740 (-)	-19.542	70.351	73.042	0.0069	0.4167	0.3	
PF3D7_1445400	PF14_0431	protein serine/threonine kinase-1 (CLK1)	2646	Pf3D7_14_v3 : 1,862,775 - 1,865,420 (-)	-19.546	64.024	60.419	0.0140	0.5389	0.3	K08287
PF3D7_1148900	PF11_0505	Plasmodium exported protein, unknown function	270	Pf3D7_11_v3 : 1,945,778 - 1,946,047 (+)	-19.549	63.846	60.439	0.0140	0.5389	0.3	
PF3D7_0911900	PFI0580c	falstatin (ICP)	1242	Pf3D7_09_v3 : 538,221 - 539,872 (-)	-19.757	66.608	80.510	0.0045	0.3757	0.3	
PF3D7_1408900	PF14_0086	tRNA-dihydrouridine synthase, putative	1023	Pf3D7_14_v3 : 351,025 - 352,047 (-)	-20.340	60.966	42.428	0.0394	0.6130	0.2	K05545
PF3D7_1402100	PF14_0023	conserved Plasmodium protein, unknown function	2640	Pf3D7_14_v3 : 81,121 - 83,760 (+)	-20.782	60.993	48.873	0.0271	0.5847	0.2	
PF3D7_1035200	PF10_0343	S-antigen	1758	Pf3D7_10_v3 : 1,394,839 - 1,396,596 (+)	-20.782	60.993	48.923	0.0270	0.5847	0.2	
PF3D7_1243700	2277.t00420, MAL12P1.418, PFL2100w	ubiquitin-conjugating enzyme E2, putative	1398	Pf3D7_12_v3 : 1,829,660 - 1,831,057 (+)	-20.983	61.229	48.551	0.0276	0.5847	0.2	
PF3D7_0408700	MAL4P2.23, PFD0430c	sporozoite micronemal protein essential for cell traversal,perforin-like protein 1 (PLP1)	2529	Pf3D7_04_v3 : 417,372 - 420,824 (-)	-21.407	69.856	92.540	0.0023	0.2481	0.2	K13834
PF3D7_1105200	PF11_0063	conserved Plasmodium protein, unknown function	1392	Pf3D7_11_v3 : 228,159 - 231,014 (-)	-22.240	61.698	58.055	0.0160	0.5551	0.2	
PF3D7_1424100	PF14_0230	60S ribosomal protein L5, putative	885	Pf3D7_14_v3 : 973,853 - 975,543 (-)	-23.026	62.252	52.297	0.0222	0.5711	0.2	K02932
PF3D7_1006400	PF10_0064	conserved Plasmodium protein, unknown function	2613	Pf3D7_10_v3 : 267,219 - 269,831 (-)	-23.221	61.467	42.782	0.0386	0.6130	0.2	

PF3D7_0512200	MAL5P1.122, PFE0605c	glutathione synthetase (GS)	1968	Pf3D7_05_v3 : 529,934 - 531,901 (-)	-23.593	57.695	41.106	0.0426	0.6304	0.2	K01920
PF3D7_0320900	MAL3P7.13, PFC0920w	histone H2A variant, putative (H2A.Z)	477	Pf3D7_03_v3 : 875,213 - 876,295 (+)	-23.593	57.600	41.108	0.0426	0.6304	0.2	K11251
PF3D7_1216900	2277.t00163, MAL12P1.163, PFL0815w	DNA-binding chaperone, putative	2820	Pf3D7_12_v3 : 670,447 - 673,266 (+)	-24.845	63.323	82.131	0.0042	0.3698	0.2	
PF3D7_1138700	PF11_0398	conserved Plasmodium protein, unknown function	5613	Pf3D7_11_v3 : 1,525,654 - 1,531,744 (-)	-25.735	58.495	52.393	0.0221	0.5711	0.2	
PF3D7_1444900	PF14_0426	conserved Plasmodium protein, unknown function	519	Pf3D7_14_v3 : 1,848,315 - 1,848,833 (+)	-25.735	58.495	52.393	0.0221	0.5711	0.2	
PF3D7_1371600	PF13_0115	erythrocyte binding like protein 1, pseudogene (EBL1)	7833	Pf3D7_13_v3 : 2,811,706 - 2,820,270 (+)	-25.735	58.710	52.393	0.0221	0.5711	0.2	
PF3D7_0415300	MAL4P1.143, PFD0740w	cdc2-related protein kinase 3 (CRK3)	4020	Pf3D7_04_v3 : 681,810 - 685,968 (+)	-25.736	58.615	52.395	0.0221	0.5711	0.2	
PF3D7_1037500	PF10_0368	dynamin-like protein (DYN2)	2130	Pf3D7_10_v3 : 1,487,959 - 1,490,088 (+)	-25.736	58.737	52.397	0.0221	0.5711	0.2	
PF3D7_0800300	MAL8P1.166, PF08_0140	erythrocyte membrane protein 1, PfEMP1 (VAR)	8943	Pf3D7_08_v3 : 40,948 - 50,939 (+)	-25.754	58.832	51.618	0.0231	0.5847	0.2	K13850
PF3D7_0936800	PFI1780w	Plasmodium exported protein (PHISTc), unknown function	1152	Pf3D7_09_v3 : 1,458,392 - 1,459,862 (+)	-25.757	58.832	51.481	0.0233	0.5847	0.2	
PF3D7_0405100	MAL4P1.49, PFD0250c	protein transport protein Sec24B (SEC24B)	4053	Pf3D7_04_v3 : 272,255 - 276,824 (-)	-26.324	58.859	49.670	0.0258	0.5847	0.2	
PF3D7_0615500	2270.t00045, MAL6P1.271, PFF0750w	cdc2-related protein kinase 5 (CRK5)	2118	Pf3D7_06_v3 : 644,052 - 646,169 (+)	-26.726	64.481	92.845	0.0023	0.2481	0.2	
PF3D7_0113000	MAL1P3.06, PFA0620c	glutamic acid-rich protein (GARP)	2022	Pf3D7_01_v3 : 487,892 - 490,127 (-)	-27.600	59.546	64.034	0.0114	0.5149	0.1	
PF3D7_0703000	PF07_0010	conserved Plasmodium protein, unknown function	5532	Pf3D7_07_v3 : 115,239 - 120,770 (+)	-27.600	59.546	64.034	0.0114	0.5149	0.1	

PF3D7_1243600	2277.t00419, MAL12P1.417, PFL2095w	translation initiation factor SUI1, putative	348	Pf3D7_12_v3 : 1,825,959 - 1,826,884 (+)	-28.913	60.123	66.084	0.0101	0.4964	0.1	K03113
PF3D7_1324800	PF13_0140	dihydrofolate synthase/folylpolyglutamate synthase (DHFS-FPGS)	1569	Pf3D7_13_v3 : 1,036,543 - 1,038,531 (-)	-29.252	60.360	75.959	0.0058	0.4167	0.1	K11754
PF3D7_1360200	MAL13P1.299	conserved protein, unknown function	774	Pf3D7_13_v3 : 2,406,838 - 2,408,385 (-)	-29.781	60.959	67.621	0.0093	0.4750	0.1	
PF3D7_1367000	MAL13P1.337	suppressor of kinetochore protein 1, putative (SKP1)	489	Pf3D7_13_v3 : 2,679,415 - 2,680,425 (-)	-30.734	61.103	88.096	0.0030	0.2914	0.1	K03094
PF3D7_1429800	PF14_0277	coatamer beta subunit, putative	4113	Pf3D7_14_v3 : 1,170,829 - 1,175,293 (+)	-30.929	61.665	73.323	0.0068	0.4167	0.1	K17301
